# Germplasm Sources, Genetic Richness, and Population Differentiation of Modern Chinese Soybean Cultivars Based on Pedigree Integrated With Genomic-Marker Analysis

**DOI:** 10.3389/fpls.2022.945839

**Published:** 2022-07-11

**Authors:** Chunyan Li, Wubin Wang, Yongpeng Pan, Fangdong Liu, Jianbo He, Chuanxiang Liu, Jiqiu Cao, Xiaoyan Zhang, Jinming Zhao, Junyi Gai

**Affiliations:** ^1^Soybean Research Institute & MARA National Center for Soybean Improvement & MARA Key Laboratory of Biology and Genetic Improvement of Soybean (General) & State Key Laboratory for Crop Genetics and Germplasm Enhancement & Jiangsu Collaborative Innovation Center for Modern Crop Production, Nanjing Agricultural University, Nanjing, China; ^2^MARA Key Laboratory of Germplasm Enhancement and Breeding Technology of Soybean, Jiaxiang, China

**Keywords:** modern Chinese soybean cultivar population (MCSCs/MCSCP), germplasm, pedigree, core-terminal ancestor, population differentiation, coefficient of parentage (COP), coefficient of genetic similarity (CGS)

## Abstract

Soybean is a native crop in China for ≈ 5,000 years. The 560 cultivars released in 2006–2015, commercialized with seeds available publicly, were collected (designated modern Chinese soybean cultivars, MCSCs), as a part of 2,371 ones released during ~100 years' breeding history. The MCSCs with their parental pedigrees were gathered, including 279, 155, and 126 cultivars from Northeast and Northwest China (NNC), Huang-Huai-Hai Valleys (HHH), and Southern China (SC), respectively. The MCSCs were tested in the field, genotyped with sequencing, and analyzed for their germplasm sources, genetic richness, and population differentiation based on pedigree integrated with genomic-marker analysis. The main results were as follows: (i) The MCSCs covering 12 of the global 13 MGs (maturity groups) showing different ecoregions with different cropping systems caused their different MG constitutions. (ii) Parental pedigree analysis showed 718 immediate parents and 604 terminal ancestors involved in MCSCs, from which 41 core-terminal ancestors were identified. (iii) NNC was richer in allele number and specific present/deficient alleles, and genetically distant from HHH and SC. (iv) The geographic grouping of MCSCs was partially consistent with marker-based clustering, indicating multiple genetic backgrounds in three eco-subpopulations. (v) Eleven major core-terminal ancestor-derived families were identified, including four derived from ancestors in NNC, four from HHH, and three from SC, containing 463 (82.68%) MCSCs with some cross-distribution among ecoregions. (vi) CGS (coefficient of genetic similarity) calculated from genomic markers showed more precision than COP (coefficient of parentage) using pedigree information in evaluating genetic relationship/differentiation. Overall, through pedigree and genomic-marker analyses, the germplasm constitutions of the three eco-subpopulations were relatively self-sufficient, and germplasm exchange is seriously required for further improvement.

## Introduction

Soybean (*Glycine max* (L.) Merr.), originated in ancient China ≈5,000 years ago (Zhao and Gai, 2004), and has disseminated all around the world as an economic crop rich in protein (~40%) and oil (~20%). In China, soybeans have been widely planted, covering almost all the arable area, all the maturity groups except MG X, and different kinds of cropping systems (Gai and Wang, 2001). Especially, during the recent decades, soybean has expanded to further higher latitudes in mono-cropping and to further early seasons in multi-cropping systems. Now, the soybean production areas in China can be roughly grouped into three major ecoregions, i.e., the Northeast and Northwest China northern spring soybean cultivation region (NNC, Northwest region was newly added), the Huang-Huai-Hai Valleys spring-summer double-cropping soybean cultivation region (HHH), and the Southern China spring-summer-autumn multi-cropping soybean cultivation region (SC).

Throughout history, ancient farmers developed soybean varieties for their own production, and a large number of farmers' varieties or landraces formed and accumulated. Along with the new landraces formed, some old ones retired. Fortunately, modern cool storage techniques saved the remained landraces. Based on the landraces, modern soybean breeders developed and released soybean cultivars scientifically, which was started in the early 20 century in China (Gai et al., 2015). In 1923–2015, altogether, 2,371 cultivars have been released in the three soybean eco-regions in China.

An effective and efficient plant breeding depends on the source of raw materials or the germplasm reservoir (gene pool) (Gai et al., 2015). In the early time, the soybean germplasm mainly included landraces and annual wild relatives (*Glycine soja* Sieb. & Zucc). With the continuous release of new cultivars used currently then turning as breeding materials for the next round of breeding, the released cultivars as well as their derived specific materials through modern technologies become a growing source of germplasm resources/reservoir, especially accompanied by the joining of various specific introductions during the breeding history. In spite of the importance of released cultivars as growing germplasm components in plant breeding, it is especially necessary to explore the phenotypic and genotypic characteristics of the released cultivars for further genetic improvement. Based on it, the germplasm/gene backgrounds and potentials, germplasm/gene deficiencies and introduction requirements, as well as new technique preparations for achieving further breeding targets may be prepared and designed in advance.

In exploring the phenotypic and genotypic characteristics of the released cultivars as the germplasm source, it has experienced three stages. The initial stage was to collect and catalog the released cultivars in addition to having the landraces collected already, with their geographical site, genetic sources, and morphological and agronomical traits evaluated and documented. In the second stage, their genetic utility was evaluated through parental pedigree analysis, for which the coefficient of parentage (COP) between/among cultivars was estimated to indicate their genetic relationship. With the rapid development of molecular biology, the third stage started by using molecular marker technology. By which the genetic relationship between/among varieties can be evaluated with a coefficient of genetic similarity (CGS) and the genetic constitution of each trait, i.e., QTL-allele or gene-allele constitutions in the population can be explored through the QTL/gene mapping procedure (Zhang et al., 2015; Meng et al., 2016; Li et al., 2017; Fu et al., 2020; Liu et al., 2021a). Based on it, the QTLs-alleles or genes-alleles can be traced in the population. This kind of study started from released cultivars and now has been extended to landraces as well as wild soybean germplasm resources. As indicated by Liu et al. (2021b), the world's 90% of soybean production is related to the soybean germplasm from Northeast China, understanding the genetic constitution of this population, in fact, is a short way to know the genetic background of the world major soybean sources.

Gai et al. (2015) summarized the pedigree analysis results as well as their germplasm bases of the 1,300 soybean cultivars released from 1923 to 2005 in China. Their work was at the second stage basically, regarding the genetic relationship through parental pedigree analysis. They showed all the parental pedigrees of 1,300 soybean cultivars released from 1923 to 2005 and traced their terminal ancestors to reveal the major germplasm sources. The cultivar improvement developed quickly after 2005 in China, there were 1,071 cultivars released at the state-/province-authorized level from 2006 to 2015. Among them, 560 were commercialized with seeds available publicly that have been collected and designated as modern Chinese soybean cultivar population (MCSCP/MCSCs). As indicated above, these recently released cultivars are historical accumulations of previous breeding efforts from 1923 through 2015. Because each round of the breeding cycle laid on the previous cultivar generations with the superior genes-alleles accumulated consecutively in the recent population. Based on this understanding, exploring the phenotypic and genotypic characteristics is in fact to understand the core germplasm of Chinese current soybean cultivars.

The present study aimed at exploring the major germplasm sources in the whole country as well as in the three ecoregions through pedigree analysis, summarizing the core ancestors of the MCSCP, and exploring the genetic richness, genetic similarity/diversity, and genetic differentiation of the MCSCP, as well as in three ecoregions through genomic marker analysis. Then, the study worked on exploring the genetic differentiation among the three ecoregions to see whether the geographic differentiation coincided with the genetic differentiation and then exploring the genetic differentiation of the MCSCP in terms of major core-terminal ancestor-derived families to see how the major terminal ancestors played their important roles in Chinese modern soybean cultivars. In addition, the COP values were compared to CGS values for their utilization in evaluating the genetic similarity/diversity among the released cultivars.

## Materials and Methods

### The Collection of the Modern Chinese Soybean Cultivars Released From 2006 to 2015

A total of 560 soybean cultivars officially released from 2006 to 2015 were collected from the public and private breeders, forming the MCSCP, including 279 from NNC, 155 from HHH, and 126 from SC (Supplementary Figure 1A). The 560 cultivars came from 26 provinces, including 122 state-authorized and 438 province-authorized cultivars (Supplementary Table 1). The passport data for all 560 cultivars can be referred to in Supplementary Table 2. The seeds of the MCSCs were collected directly from the respective breeders/institutions, while their parental pedigrees were mainly collected from the breeders as well as publications.

### Field Experiments

In 2018, the 560 cultivars were tested in one-row plots, with a plot length of 1 m, and a row space of 0.4 m, in a randomized blocks design with two replications at the Dangtu experimental station of Nanjing Agricultural University in Anhui province, China, and three replications at Jiaxiang experimental station of the Key Laboratory of Germplasm Enhancement and Breeding Technology of Soybean in Shandong province, China. The sowing date was June 26 at Dangtu and June 23 at Jiaxiang. The evaluated traits were: beginning bloom (R1) and full maturity (R8) according to Fehr and Caviness (1977), days to flowering and days to maturity calculated as the days from sowing to R1 and R8, respectively, main stem node number counted at maturity with the cotyledonary node as zero up to the top, 100-seed weight evaluated three times per plot, and protein content and oil content evaluated per plot base using FOSS InfratecTM1241 (Hoganas, Sweden).

### Statistical Analysis

PROC MEANS in SAS/STAT 9.4 (SAS Institute Inc., Cary, NC, USA) was used to calculate the descriptive statistics (mean, minimum, and maximum) of days to flowering, days to maturity, main stem node number, 100-seed weight, seed protein content, and seed oil content. PROC GLM was used for the analysis of variance (ANOVA), the linear model is


$$\begin{array}{l} y_{ijk}\;=\;\mu +t_{i}+b_{j\left(i\right)}+g_{k}+{\left(gt\right)}_{ik}+\varepsilon_{ijk} \end{array}$$

where μ is the population mean; *t*_*i*_ is the effect of *i*th environment; *b*__*j*_(i)_ is the effect of *j*th block within the *i*th environment; *g*_*k*_ is the effect of *k*th genotype and (*gt*)_*ik*_ is the genotype-by-environment interaction effect; ε_*ijk*_ is the random error and ε_*ijk*_ ~ *N*(0, σ^2^). The variance components were estimated according to the expected mean squares in ANOVA. The genetic coefficient of variation was calculated as *GCV* = σ_*g*_/μ × 100%, where σ_*g*_ is the square root of genotypic variance.

The maturity group of the 560 soybean cultivars was determined with the growth period record obtained from the field experiments according to the standards in the literature (Fu et al., 2016; Tang et al., 2016; Liu et al., 2017; Song et al., 2019).

### Parental Pedigree, Coefficient of Parentage, and Core-Terminal Ancestor-Derived Family

**Figure 1:**
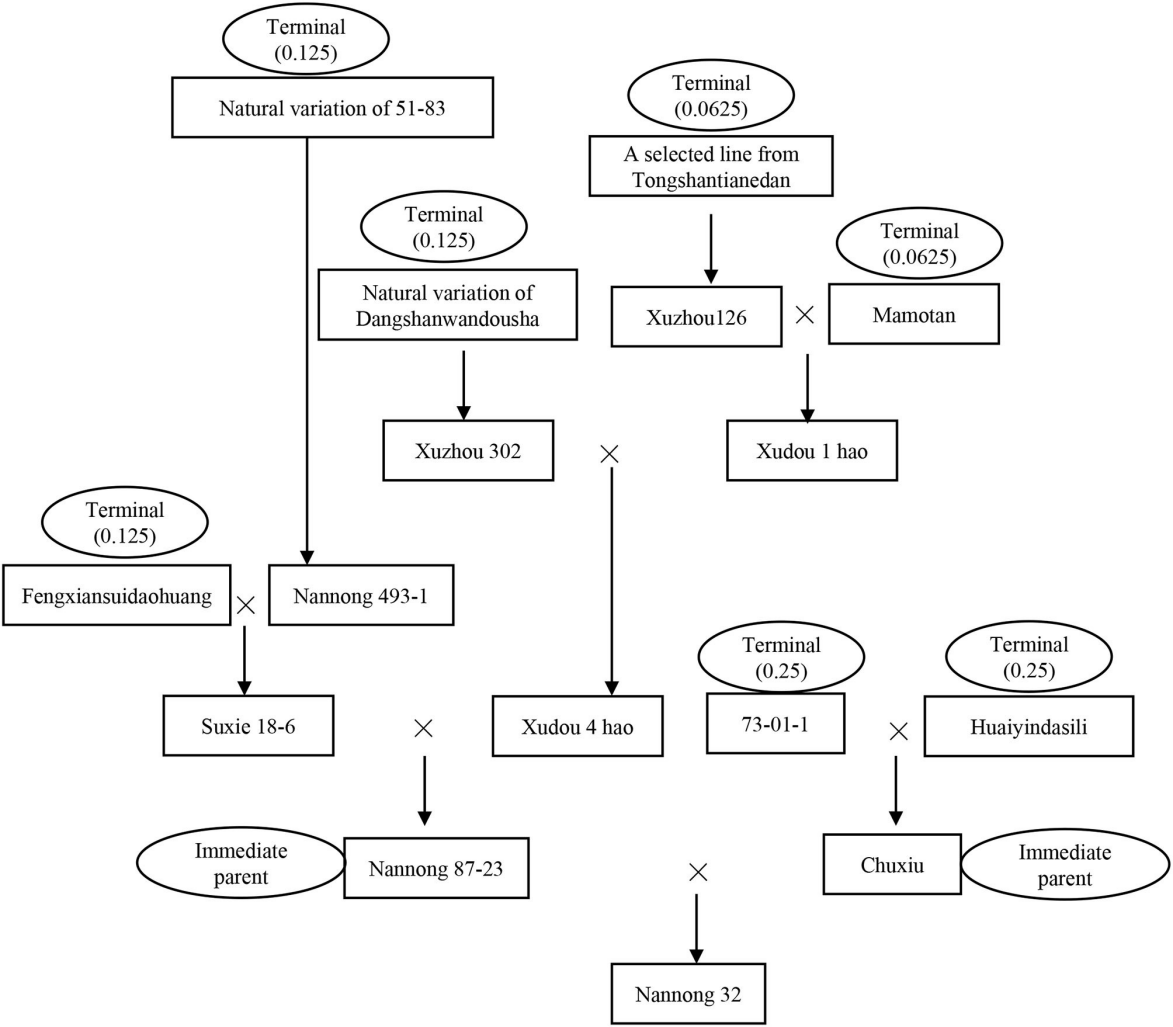
The parental pedigree of the released cultivar Nannong 32. Nannong 87-23 and Chuxiu were the immediate female parent and male parents of Nannong 32, respectively. We define the ultimate landrace or breeding lines/cultivars that can no longer trace their genetic origin as a terminal ancestor. A total of seven terminal ancestors have been traced for Nannong 32, the nuclear genetic contribution value of 73-01-1, Huaiyindasili, Fengxiansuidaohuang, 51-83, Dangshanwandousha, Tongshantianedan, and Mamotan were 0.25, 0.25, 0.125, 0.125, 0.125, 0.0625, and 0.0625, respectively, which were added to 1. The cytoplasmic terminal ancestor of Nannong 32 was traced to Fengxiansuidaohuang, whose cytoplasmic genetic contribution value was 1.

The method of parental pedigree analysis in this study was the same as that indicated by Gai et al. (1998) and Xiong et al. (2008). In pure line selection or mutation breeding, the nuclear genetic contribution value of the source cultivar/variety/line to the pure line or mutant is recorded as 1. In hybridization breeding, the nuclear genetic contribution values of both the female and male parents to the hybrids are 1/2 or 0.5, the same proportion for the parents of the last hybridization, and up to terminal parents/ancestors, in this way, the total nuclear contribution of all parental ancestors to the derived cultivar must be 1. As for the cytoplasmic contribution, the terminal female ancestor of the immediate parent of the last round of hybridization contributes 1, while the others are zero. These roles are used in all kinds of crossing-types, such as the example of Nannong 32 in Figure 1, where the nuclear contribution values of the seven terminal ancestors in different crossing cycles to Nannong 32 were marked and added to 1. While the immediate cytoplasm parent Nannong 87-23 provided cytoplasm to Nannong 32, which can be traced to the terminal cytoplasm parent, a landrace of Fengxiansuidaohuang, to Nannong 32, so the cytoplasm contribution value of Fengxiansuidaohuang is 1.

The coefficient of parentage (COP) was calculated referring to Cox et al. (1985) and Cui et al. (2000). The basic rules are as the following: (i) A cultivar derived from hybridization obtained 50% of its germplasm from each parent. (ii) All terminal ancestors, parents, and derived cultivars or breeding lines were homozygous and homogeneous. (iii) The COP value of all terminal ancestors is 0. (iv) The COP value between the cultivar and its selected line and between a naturally occurred or mutagen-induced mutation and its antecedent are 0.75. (v) The COP value between the cultivars and itself is 1. (vi) The calculation formula of COP value between cultivars containing some of the same parents, is COP_SD_ = Σ[ (1/2)^*n*^], where COP represents the coefficient of parentage between cultivar S and cultivar D, *n* represents the number of generations from the common parents to cultivar S and cultivar D. The population COP matrix was established based on all COP values among cultivars.

According to the geographic origin of the terminal ancestors of MCSCs, all the terminal ancestors were grouped into four sets NNC, HHH, SC, and foreign introduction. Those terminal ancestors of each set with three or more breeding cycles were picked up as candidate terminal ancestors, from which, those with two or more of the four indicators higher than the average of the candidate terminal ancestors were nominated as population-level core-terminal ancestors of the MCSCP. The four indicators were nuclear genetic contribution value, cytoplasmic genetic contribution value, number of derived cultivars, and the number of breeding cycles involved. In the core-terminal ancestors of the MCSCP, those involved in an eco-region are also the eco-regional core-terminal ancestors.

Based on the number of derived cultivars, the nuclear genetic contribution, and the cytoplasmic contribution of the core-terminal ancestors, 4, 4, and 3 major core-terminal ancestors with their derived cultivars were recognized as the major core-terminal ancestor-derived families in NNC, HHH, and SC, respectively.

### Genotyping of the MCSCP and Assembly of Multi-Haplotype Genomic Markers

The whole-genome sequencing (WGS) was performed at Annoroad Ltd., Beijing, China. The total DNA samples of the 560 released cultivars were extracted from their young leaves using the CTAB method (Murray and Thompson, 1980). The sequences were obtained using Illumina HiSeq 2000 instrument through the multiplexed shotgun genotyping method (Andolfatto et al., 2011) with DNA fragments between 400 and 600 bp, generating 8.42 billion paired-end reads of 150 bp (1262.66 Gb of sequence), ≈2.30 × in depth and 73.7% coverage. All sequence reads were aligned against the reference genome Wm82.v2 (Schmutz et al., 2010) using BWA (Li, 2013). The GATK software (McKenna et al., 2010) was applied for population SNP calling. The SNPs of the 560 cultivars were polymorphic with a missing rate ≤ 20%, heterozygous rate ≤ 10%, and minor allele frequency (MAF) ≥ 0.01. The Beagle software (Browning et al., 2018) was used for genotyping SNP imputation after heterozygous alleles were turned into missing alleles.

The whole-genome sequence was divided at a certain LD value (*D*′ > 0.7) into SNP linkage disequilibrium blocks (SNPLDBs) as genomic markers with their multiple haplotypes/alleles using the RTM-GWAS software according to He et al. (2017). In the MCSC population, 277,581 SNPs were identified and further organized into 28,066 SNPLDBs. The SNP distribution on chromosomes of the MCSC population was shown in Supplementary Figure 1B.

### Genetic Richness, Similarity, and Differentiation of the MCSCP

The genetic diversity of the MCSCP was evaluated in two aspects: genetic richness (or the number of different alleles) and allele frequency dispersion on a locus. In the present study, the SNPLDB markers with their haplotypes were used to evaluate the genetic diversity, here the number of different haplotypes represents genetic richness while allele frequency dispersion was calculated (Carlson et al., 2005),


$$\begin{array}{l} \pi \;=\;\frac{n}{n-1}\overset{q}{\underset{i\;=\;1}{\sum }}p_{i}\left(1\;-\;p_{i}\right), \end{array}$$

where *n* is the capacity of the population, *p*_*i*_ is the frequency of the *i*th allele/haplotype on a locus, *q* is the total allele number. The π of a SNPLDB was calculated based on the frequency of alleles in SNPLDBs, then the average of π of all SNPLDB in the population was used to represent the genetic dispersion of the population.

Wen et al. (2009) defined the SPA (specific present allele) of a population as an allele present in the population and SDA (specific deficient allele) as an allele present in all other populations but absent in the population. These two indicators were used to evaluate the specificity of the three subpopulations.

Genetic similarity (*s*_*ij*_)/distance (*d*_*ij*_) was calculated for all pairs of cultivars: *d*_*ij*_ = 1 – *s*_*ij*_, where *s*_*ij*_ is the coefficient of genetic similarity (CGS), calculated as $s_{ij}\;=\;\overset{m}{\underset{k\;=\;1}{\sum }}C_{ijk}/\;\left(2m\right)$, where *C*_*ijk*_ is the common allele number of the *i*th and *j*th cultivar at the *k*th SNPLDB marker, and m is the total number of SNPLDB markers. The CGS matrix was calculated based on all SNPLDBs using the RTM-GWAS software (He et al., 2017). The R software package (ECOdist) was used for the Mantel test to analyze the correlation between COP matrice and CGS matrice of the MCSC population (Goslee and Urban, 2007).

Principal component analysis was performed using RTM-GWAS (He et al., 2017) based on SNPLDB of the MCSCP. The first three principal components were used to analyze the relationship of population structure among three subpopulations of China.

The differentiation coefficient *F*_*ST*_ statistics between two subpopulations were calculated using the VCFtools software (Danecek et al., 2011) through the formula proposed by Weir and Cockerham (1984).


$$\begin{array}{l} F_{ST}=\frac{\underset{u}{\sum }a_{u}}{\underset{u}{\sum }\left(a_{u}+b_{u}+c_{u}\right)}, \end{array}$$

where *a*_*u*_, *b*_*u*_, and *c*_*u*_ are the variances of frequencies for the *u*th allele between subpopulations, between individuals within subpopulations, and between gametes within individuals, respectively.

### Genetic Clustering

Genetic clustering was performed using the genetic distance matrix of *d*_*ij*_ for both the 560 cultivars and the 11 major core-terminal ancestor-derived families using the neighbor-joining procedure of MEGA X (Kumar et al., 2018). The neighbor-joining tree of the 560 cultivars was generated from Figtree version 1.4.3 (http://tree.bio.ed.ac.uk/software/figtree/).

## Results

### Agronomic Characteristics of the Modern Chinese Soybean Cultivars, Their Variability, and Differentiation Among Three Eco-Regions in China

Under the uniform environment testing at Dangtu and Jiaxiang, China, respectively, the MCSCP was performed to cover MG 000–MG IX (except MG X). All the six agronomic traits showed very significant variation with its DTF (days to flowering) ranging from 23.20 to 82.75 d, DTM (days to maturity) ranging from 63.25 to 144 d, and MSN (main stem node number) in 7.38–27.90 nodes, 100-seed weight in 7.17–40.26 g, seed protein content in 34.8–49.53% and seed oil content in 16.4–25.2% averaged over the two locations (Table 1). The results of the analysis of variance showed that there were significant differences among cultivars, environments, and interactions between cultivars and environments in the MCSCP (Supplementary Table 3).

**Table 1:** Variability of agronomic traits within/among NNC, HHH, and SC of the MCSCP tested in Jiaxiang and Dangtu, China.

**Ecoregion**	**No. of cultivars**	**Days to flowering**			**Days to maturity**			**Main stem node number**		
		**Mean (d)**	**Range (d)**	**GCV (%)**	**Mean (d)**	**Range (d)**	**GCV (%)**	**Mean (node)**	**Range (node)**	**GCV (%)**
NNC	279	27.64c	23.20–38.60	11.09	87.62c	63.25–105.00	11.23	12.80c	8.32–19.36	15.18
HHH	155	36.15b	26.20–44.20	9.85	99.05b	90.80–114.25	2.15	15.36b	10.48–21.26	13.57
SC	126	47.23a	28.00–82.75	25.33	111.68a	89.40–144.00	12.05	16.31a	7.38–27.90	20.88
MCSCP	560	34.42	23.20–82.75	28.74	96.34	63.25–144.00	13.85	14.31	7.38–27.90	19.64
**Ecoregion**	**Maturity group (MG)**	**100-seed weight**			**Seed protein content**			**Seed oil content**		
		**Mean (g)**	**Range (g)**	**GCV (%)**	**Mean (%)**	**Range (%)**	**GCV (%)**	**Mean (%)**	**Range (%)**	**GCV (%)**
NNC	000–IV	16.73c	7.17–25.07	13.13	41.59b	36.20–48.88	4.66	22.38a	17.20–25.20	4.76
HHH	I–V	18.28b	13.88–24.56	5.47	41.59b	36.94–47.06	3.90	21.31b	18.85–24.16	4.74
SC	I–IX	19.86a	12.25–40.26	13.82	43.31a	34.80–49.53	5.36	19.58c	16.40–22.68	3.97
MCSCP	000–IX	17.83	7.17–40.26	13.38	41.97	34.80–49.53	6.79	21.45	16.40–25.20	6.32

*Ecoregion: NNC, HHH, and SC represent Northeast and Northwest China, Huang-Huai-Hai Valleys, and Southern China, respectively. MCSCP means the modern Chinese soybean cultivar population released in 2006–2015*.

*MG, maturity group; GCV, genotypic coefficient of variation*.

*Different letters of a, b, and c in Column Mean indicate the means are significant at p ≤ 0.01*.

**Figure 2:**
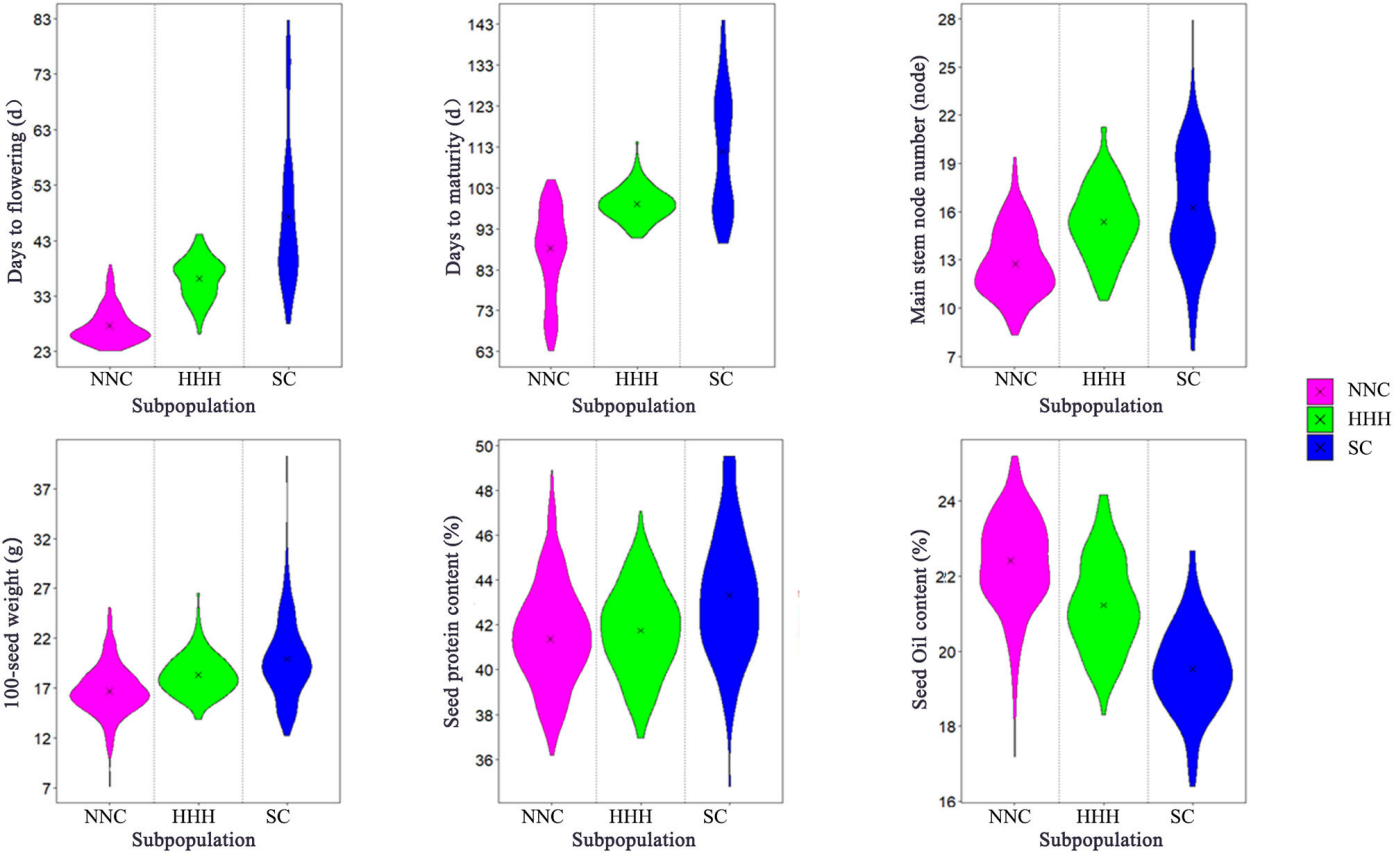
Violin diagram of six agronomic traits of NNC, HHH, and SC. NNC, HHH, and SC represent Northeast and Northwest China, Huang-Huai-Hai Valleys, and Southern China, respectively.

These agronomic traits varied among the three eco-regional subpopulations, especially those in SC under multiple cropping systems, its maturity groups covering MG I–IX, wider than the other two subpopulations (MG 000-IV in NNC and MG I–V in HHH). Its average DTF and DTM were 47.23 d (ranging from 28 to 82.75 d) and 111.68 d (ranging from 89.4 to 144 d), respectively, longer than the other two subpopulations (Table 1, Figure 2). Its average MSN and 100-seed weights was 16.31 nodes (ranging in 7.38–27.9 nodes) and 19.86 g (ranging in 12.25–40.26 g), more than the other two subpopulations. Its average protein content (43.31%, ranging from 34.8 to 49.53%) was significantly higher while the oil content (19.58%, ranging from 16.4 to 22.68%) was obviously lower than the other two subpopulations (Table 1, Figure 2). On the other hand, in the modern released cultivars in the HHH ecoregion, the maturity group varied from MG I to MG V, fitted in the double cropping system, while for those in the NNC region the MG varied in MG 000–MG IV, fitted in their full-season single cropping system. The different geographic locations caused the different cropping systems, in turn, caused the different maturity group constitutions in the three soybean ecoregions. For the agronomic traits, the growth-related traits, MG, DTF, DTM, and MSN, varied especially wide among the geographic regions due to the systematically changed photo-thermal conditions. While for the seed traits, 100-seed weight, protein content, and oil content varied with the selection pressure from farmers and breeders, in which, 100-seed weight varied wider than the other two traits because artificial selection pressure could act on seed size by sight in the long history.

### Germplasm Sources and Core-Terminal Ancestors of Modern Chinese Soybean Cultivars Based on Pedigree Analysis

The genetic background of the MCSCP was traced, starting from establishing the parental pedigree of an individual cultivar, from which, the immediate parents recognized, in turn, the terminal ancestors of a cultivar, the core-terminal ancestors on the whole population-level and ecoregion-level identified.

**Figure 3:**
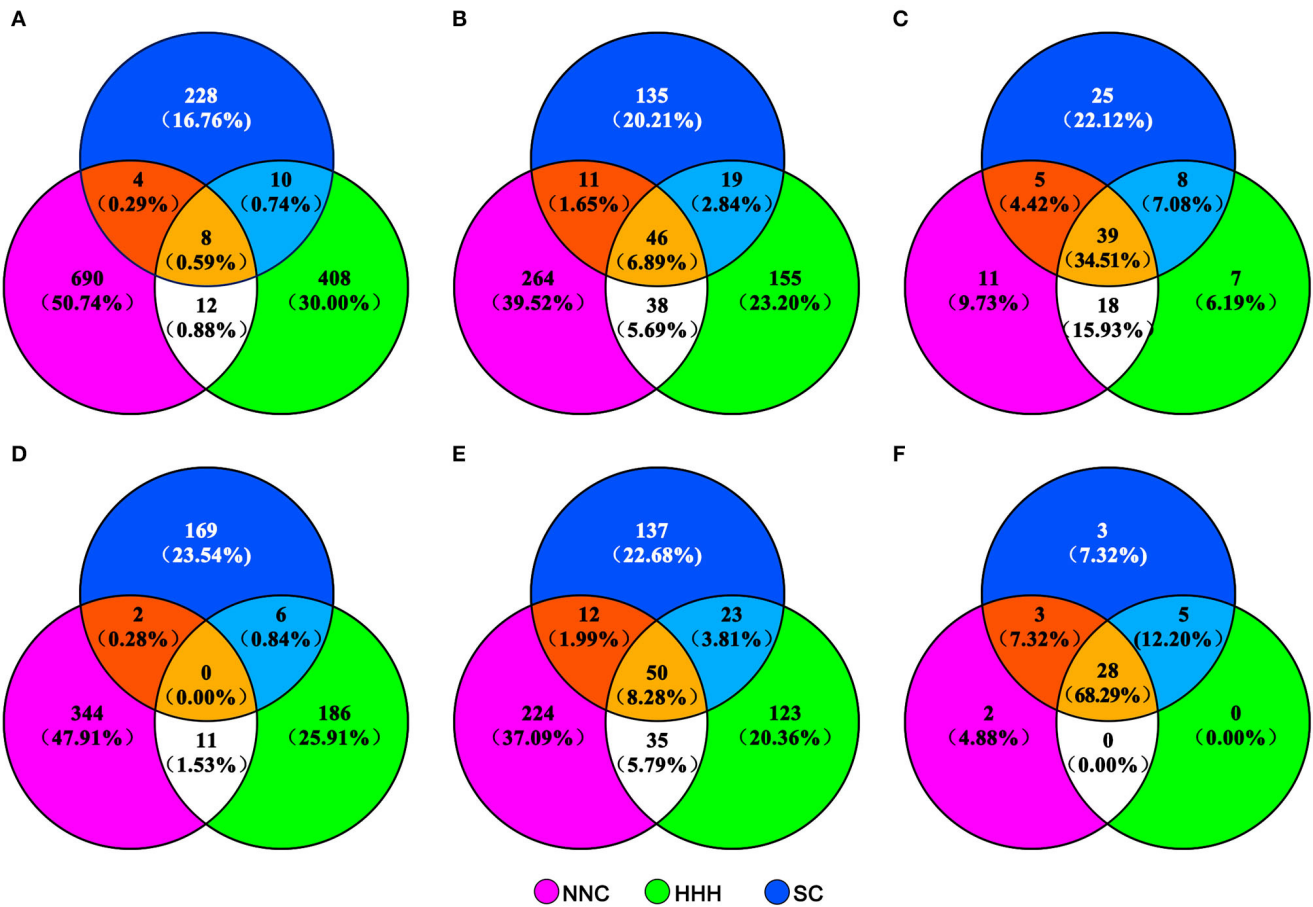
Venn diagram of different parent/ancestor types of cultivars released in NNC, HHH, and SC in different periods. NNC, HHH, and SC represent cultivars released in Northeast and Northwest China, Huang-Huai-Hai Valleys, and Southern China, respectively. The distribution of immediate parents, total terminal ancestors, and core terminal ancestors of cultivars released in 1923–2005 in the three ecoregions are in **(A–C)**, while those released in 2006–2015 are in **(D–F)**, respectively.

Nearly all cultivars (93.39%) of the MCSCP were developed through hybridization breeding (Supplementary Table 4). A total of 718 immediate parents were involved in MCSC development (Figure 3D), of which 74.09% were used only once. Only 17 direct parents were used more than six times (Supplementary Table 5). The major immediate parental types of the three subpopulations were cultivars and breeding lines, and the proportion of landrace and foreign variety as immediate parents in SC was significantly more than in the other two subpopulations (Supplementary Table 5). Compared to the proportion of immediate parents of cultivars released in 1923–2005 in the three regions, the proportion of immediate parents used only in southern cultivars was increased by 6.78%, while decreased by 4.09 and 2.83% in HHH and NNC, respectively, and the proportion of immediate parents shared by two or three regions changed slightly (Figures 3A,D).

The 560 cultivars were traced back to 604 terminal ancestors, of which 50 were shared by the three subpopulations and 70 were shared by two subpopulations (Figure 3E). Compared to those in the three regions released in 1923–2005, the proportion of the terminal ancestors used uniquely in their respective local ecoregions was about the same as in 1923–2005, and those shared by two or three regions increased (Figures 3B,E).

For the MCSCP, a total of 41 whole population-level core-terminal ancestors were identified from the 604 terminal ancestors, depending on their utilization in multiple breeding cycles, derived number of cultivars, nuclear contribution, and cytoplasmic contribution in the population as indicated above (Table 2). Their names with corresponding codes are shown in Supplementary Table 6. As an example, in Nannong 32 in Figure 1, there are two immediate parents and seven terminal ancestors, the nuclear contribution value of the seven terminal ancestors was added to 1 and the cytoplasmic contribution value was 1 from Fengxiansuidaohuang (CA-27). Among the seven terminal ancestors, three ones, Fengxiansuidaohuang (CA-27), 51-83 (CA-28), and Tongshantianedan (CA-16) are population-level core-terminal ancestors (Figure 1, Table 2). The 41 population-level core-terminal ancestors, accounting for 6.79% of the total terminal ancestors, contributed 44.47% to the total nuclear genetic contribution and 54.47% to the total cytoplasmic genetic contribution of the MCSCP (Supplementary Table 7). These population-level core-terminal ancestors comprise 14 ones that originated from NNC, 11 from HHH, nine from SC, and seven from abroad (Table 2, Supplementary Table 6). Compared to the population-level core-terminal ancestors of soybean cultivars released between 1923 and 2005 (Xiong et al., 2007), all 41 core ancestors are included in the 113 previous population-level core-terminal ancestors but varied in their genetic contribution values. The genetic contribution changes of the core-terminal ancestors might be due to the shift of breeding objectives and the infiltration of new ones.

**Table 2:** The core-terminal ancestors of the MCSCP as well as NNC, HHH and SC subpopulations.

**Code**	**MCSCP**			**NNC**			**HHH**			**SC**		
	**NGC(%) a**	**CGC(%) b**	**No. C**	**NGC (%)**	**CGC (%)**	**No. C**	**NGC (%)**	**CGC (%)**	**No. C**	**NGC (%)**	**CGC (%)**	**No. C**
CA-01	3.04 (3.52)	4.46 (6.38)	195	6.02	8.96	190	0.04		2	0.15		3
CA-02	2.56 (5.05)	0.00 (1.31)	296	4.78		233	0.48		47	0.19		16
CA-03	2.41 (4.46)	10.18 (10.38)	272	4.49	20.43	225	0.39		38	0.26		9
CA-04	2.05 (1.83)	0.00 (0.08)	160	4.06		157	0.03		1	0.07		2
CA-05	2.04 (2.78)	3.57 (3.54)	220	3.15	7.17	150	1.16		50	0.66		20
CA-06	1.66 (1.55)	5.18 (2.00)	109	2.84	9.68	87	0.42	0.65	15	0.57	0.79	7
CA-07	1.31 (1.23)		190	2.12		126	0.45		44	0.57		20
CA-08	1.17 (2.22)	0.00 (0.08)	191	1.96		140	0.44		36	0.31		15
CA-09	1.04 (0.89)	5.00 (4.46)	101	2.02	10.04	98	0.04		1	0.11		2
CA-10	0.85 (1.08)	0.18 (0.54)	82	1.62		81				0.2	0.79	1
CA-11	0.80 (0.78)		75	1.56		73				0.11		2
CA-12	0.71 (0.51)	0.00 (0.31)	96	1.43		96						
CA-13	0.65 (0.47)	0.00 (0.08)	51	1.31		51						
CA-14	0.36 (0.62)	0.00 (0.46)	118	0.38		73	0.39		31	0.27		14
CA-15	2.41 (2.87)	4.64 (3.92)	144	0.14	0.72	5	6.94	12.26	110	1.9	3.97	29
CA-16	1.12 (1.38)	0.18 (0.92)	127	0.02		2	3.43	0.65	101	0.72		24
CA-17	1.06 (0.57)	2.50 (1.23)	44				3.63	8.39	40	0.24	0.79	4
CA-18	0.98 (0.52)		64	0.09		1	3.26		59	0.15		4
CA-19	0.89 (1.63)	1.25 (2.15)	96	0.1		9	2.72	4.52	74	0.38		13
CA-20	0.82 (1.48)	1.61 (2.92)	113	0.17	0.36	13	2.22	5.16	83	0.53		17
CA-21	0.77 (0.43)	3.04 (2.00)	70	0.05		2	2.56	10.97	62	0.19		6
CA-22	0.75 (0.49)	0.00 (0.08)	75	0.05		2	2.46		67	0.22		6
CA-23	0.52 (0.33)	0.71 (0.92)	63	0.04		1	1.75	1.94	58	0.09	0.79	4
CA-24	0.39 (0.64)		107	0.09		13	1.06		81	0.23		13
CA-25	0.39 (0.77)	0.00 (0.23)	107	0.09		13	1.06		81	0.23		13
CA-26	1.36 (1.48)	3.39 (3.08)	32							6.05	15.08	32
CA-27	0.80 (0.98)	1.61 (3.46)	46				1.14	1.29	33	2.15	5.56	13
CA-28	0.52 (0.77)	0.71 (3.15)	43				1.16	1.94	36	0.91	0.79	7
CA-29	0.49 (0.72)	1.61 (2.62)	19	0.04	0.36	1				2.1	6.35	18
CA-30	0.31 (0.32)	0.00 (2.23)	28				0.96		23	0.19		5
CA-31	0.27 (0.10)	0.89 (1.31)	32				0.98	3.23	31	0.01		1
CA-32	0.16 (0.14)	0.00 (0.46)	8							0.69		8
CA-33	0.03 (0.22)	0.36 (0.62)	3							0.15	1.59	3
CA-34	3.16 (3.13)	0.36 (0.38)	186	6.22	0.72	181	0.2		4	0.05		1
CA-35	1.66 (1.80)	0.00 (0.46)	220	2.37		135	1.15		58	0.74		27
CA-36	1.19 (1.21)	3.04 (1.69)	135	0.45	0.72	44	2.82	9.68	77	0.83		14
CA-37	1.15 (1.14)		156	0.47		48	2.58		79	0.9		29
CA-38	0.79 (0.61)		184	1.08		124	0.76		53	0.21		7
CA-39	0.72 (0.94)	0.00 (0.08)	179	1.1		126	0.37		32	0.3		21
CA-40	0.56 (0.71)		127	0.01		2	1.72		101	0.36		24
CA-41	0.56 (0.71)		127	0.01		2	1.72		101	0.36		24
Total	44.47 (53.08)	54.47 (63.53)		50.33	59.16		50.49	60.68		24.35	36.50	

*Code: the codes of core-terminal ancestors, and their corresponding names were shown in Supplementary Table 6*.

*MCSCP is the abbreviation for the modern Chinese soybean cultivar population. NNC, HHH, and SC represent cultivars released in Northeast and Northwest China, Huang-Huai-Hai Valleys, and Southern China, respectively*.

*NGC: the percentage of nuclear genetic contribution relative to the total released cultivars in the population; CGC: the percentage of cytoplasmic genetic contribution relative to the total released cultivars in the population; No. C: number of derived cultivars*.

*^a^ and ^b^: in Column NGC and CGC, those in parentheses are the percentage of nuclear genetic contribution and cytoplasmic genetic contribution in the population (1300 released cultivars) released in 1923–2005, respectively*.

Among the 41 core-terminal ancestors, 33, 33, and 39 core-terminal ancestors are involved in NNC, HHH, and SC subpopulations, respectively, while 28 core terminal-ancestors are shared by all the three subpopulations (Table 2, Figure 3F). In the NNC ecoregion, about 1/4 of the nuclear genetic contribution came from the top five core terminal-ancestors (CA-01,−02,−03,−04, and−34), and more than half of cytoplasmic genetic contribution from the top five core terminal-ancestors (CA-01,−03,−05,−06, and−09), which are important terminal-ancestors in this region. In the HHH ecoregion, about 1/5 of nuclear genetic contribution from the top five core terminal-ancestors (CA-15,−16,−17,−18, and−36) and nearly half cytoplasmic genetic contribution from the top five core terminal-ancestors (CA-15,−17,−20,−21, and−36), which are important terminal-ancestors in this region. In SC ecoregion, about 1/10 of nuclear genetic contribution from the top five core terminal-ancestors (CA-15,−26,−27,−28, and−29) and nearly half cytoplasmic genetic contribution from the top five core terminal-ancestors (CA-15,−26,−27,−29, and−33), which are important terminal-ancestors in this region (Table 2). Compared to the proportion of whole population-level core terminal-ancestors of cultivars released in 1923–2005 in the three ecoregions, the proportion of core-terminal ancestors monopolized in SC, and the proportion of core-terminal ancestors shared by NNC and HHH decreased greatly, while those shared by NNC, HHH, and SC increased greatly (Figures 3C,F). Compared to the proportions of the immediate parents, terminal ancestors, and core-terminal ancestors in the three ecoregions from 1923 to 2005, those in NNC and HHH decreased, and those shared by HHH and SC increased to different degrees, indicating that in the breeding period of 2006–2015, the germplasm from HHH and NNC ecoregions contributed to the released cultivars in SC.

### Genetic Richness and Differentiation of the MCSCP Based on Genomic Marker Analysis

Based on whole-genome sequencing, a total of 28,066 SNPLDBs with their 84,069 haplotypes were assembled and used in the genetic constitution analysis of the MCSCP with an SNPLDB-haplotype looked like a locus-allele. The total genetic richness (or the total number of alleles) of the MCSCP was 84,069 alleles. While the genetic richness of NNC, HHH, and SC subpopulations was 74,386 (88.48%), 72,894 (86.71%), and 72,843 (86.65%) alleles on the 28,066 loci, each locus with 2.65, 2.60, and 2.60 alleles, ranging in 2–19, 2–17, and 2–15 alleles per locus, respectively, indicating NNC with more genetic richness than the others (Table 3). Correspondingly, the allele-load per cultivar was 266.62 (74,386/279), 470.28 (72,894/155), and 578.12 (72,843/126) in NNC, HHH, and SC, respectively, implying more genetic difference among cultivars in latter ecoregions. On the other hand, the genetic dispersion among alleles on a locus (π) of NNC (0.106) was lower than that of HHH (0.129), and in turn lower than that of SC (0.146), which means more allele frequency dispersion on the locus in HHH, and further in SC (Table 3).

**Table 3:** Genetic richness, dispersion, similarity, and differentiation of NNC, HHH, and SC subpopulations in the MCSCP.

**Subpopulation/ Population**	**No. of cultivars**	**Allele No**.			**π**		**SPA**	**SDA**	**Shared allele** **(%)**		
		**Total**	**Per locus**	**Max/Locus**	**Mean**	**Max**			**NNC**	**HHH**	**SC**
NNC	279	74,386	2.65	19	0.106	0.889	4,603	6,729			
HHH	155	72,894	2.60	17	0.129	0.876	560	4,198	80.28		
SC	126	72,843	2.60	15	0.146	0.900	2,374	6,061	76.30	83.39	
MCSCP	560	84,069	3.00	21	0.128	0.909	7,537	16,988			
**Subpopulation/ Population**	**COP (×100)**				**CGS** **(×100)**				**F**_**ST**_ **(×100)**		
	**NNC**	**HHH**	**SC**		**NNC**	**HHH**	**SC**		**NNC**	**HHH**	**SC**
NNC	2.74				89.35						
HHH	0.23	2.11			87.32	87.08			8.38		
SC	0.12	0.37	1.15		86.12	85.66	85.35		10.45	4.25	

*Subpopulation/Population: MCSCP represents the modern Chinese soybean cultivar population released in 2006–2015. NNC, HHH, and SC represent cultivars released in Northeast and Northwest China, Huang-Huai-Hai Valleys, and Southern China, respectively*.

*SPA, specific-present allele; SDA, specific-deficient allele*.

*Shared allele: the percentage of shared alleles to all alleles in the two population*.

*COP: coefficient of parentage within and between NNC, HHH, and SC subpopulations, the actual values were multiplied by 100 in the table*.

*CGS: coefficient of genetic similarity within and between NNC, HHH, and SC subpopulations, the actual values were multiplied by 100 in the table*.

*F_ST_: fixation index between NNC, HHH, and SC subpopulations, the actual values were multiplied by 100 in the table*.

To measure the genetic differentiation among subpopulations, the specific-present and specific-deficient alleles are substantial indicators. Here, a total of 7,537 SPAs and 16,988 SDAs were detected in the MCSCP, in which HHH had the smallest number of SPA (560) and SDA (4,198), many less than those in SC (2,374 and 6,061), and in turn less than those in NNC (4,603 and 6,729). The shared alleles between HHH and NNC (80.28%) and between HHH and SC (83.39%) were more than that between NNC and SC (76.3%), indicating genetically NNC was more distant from the other two subpopulations. It might be due to the single cropping system in NNC while double cropping in HHH and multiple cropping in SC (Table 3).

The genetic differentiation among ecoregions may be measured also using genetic similarity (*s*_*ij*_) because the genetic distance is in fact *d*_*ij*_ = 1 – *s*_*ij*_. Here we use average CGS to measure the population genetic differentiation, NNC was less distant to HHH (more similar as 87.32%) in comparison to SC (86.12%) and less distant to that between HHH and SC (85.66%). While in COP from pedigree analysis (larger COP means smaller genetic differentiation) and coefficient of differentiation *F*_*ST*_ with the allele frequency included (larger *F*_*ST*_ means larger genetic differentiation), the results indicated that the genetic differentiation between HHH and NNC was lower (0.23 and 8.38%) than that between NNC and SC (0.12 and 10.45%). However, the differentiation between HHH and SC was the lowest (0.37 and 4.25%), which is somewhat different from the results in CGS that high differentiation in CGS but low differentiation in COP and *F*_*ST*_ (Table 3).

In summary, from the genetic constitution analysis, the NNC subpopulation was richer in allele number and SPA/SDA alleles, and genetically more distant from the other two subpopulations. The relationship among the three subpopulations was just as the principal component analysis showed that the cultivars of NNC (with a wide variation) connected with those of HHH and then extended to SC (Supplementary Figure 2).

### Genetic Differentiation of the MCSCP in Terms of Genetic Clustering

**Figure 4:**
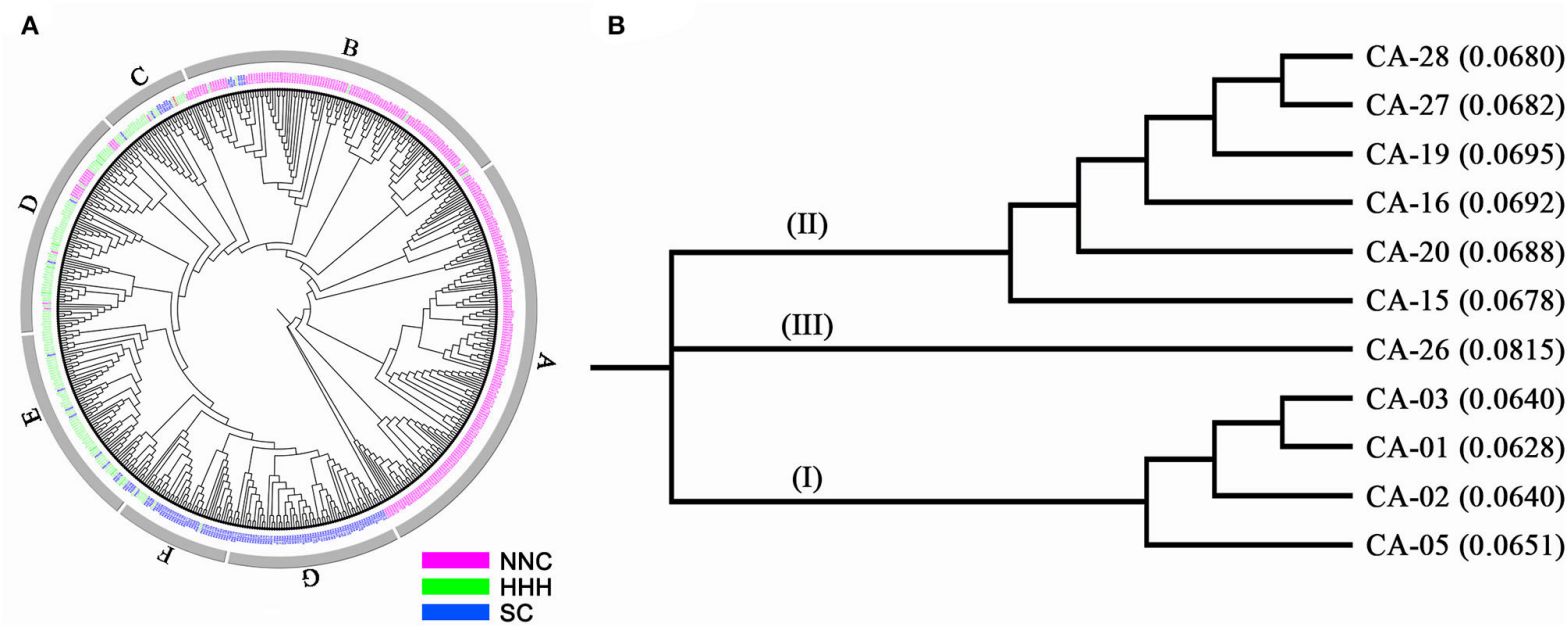
Neighbor-joining clustering of the MCSCP and 11 major core-terminal ancestor-derived families. **(A)** The neighbor-joining tree of MCSCP, A–G were names of each neighbor-joining group. Cultivars from NNC, HHH, and SC are shown in three colors. **(B)** The neighbor-joining tree of 11 major core-terminal ancestor-derived families. The distance to the root is in the parenthesis.

The MCSCP differentiated among geographic regions, whether this kind of differentiation coincided with genetic differentiation was checked through the Neighbor-joining cluster analysis. The 560 cultivars were clustered into seven groups designated as A, B, to G (Figure 4A, Table 4). The NNC cultivars were clustered in A, B, C, and D groups; while the HHH cultivars were in B to F groups and SC cultivars were in B to G groups. The three geographic subpopulations had a different distribution among the genetic clusters which means the subpopulations had their major-specific cluster(s) but shared some clusters. All 150 cultivars in Group A were from NNC, while most of the cultivars in B (108) were also from NNC with the others from HHH and SC. In C, D, and E groups, the major ones were from HHH, while some in C, and D form NNC and the others from SC. In the F group, the major ones were from SC while the others were from HHH; but all the 62 cultivars in the G group were purely from SC. From the above, the clustering results indicated the geographic grouping was not completely but partially consistent with the genetic clustering. Therefore, in each subpopulation, there exist different genetic sources. This understanding also coincided with the geographic distribution of the ancestor-derived Cultivar families in the subsequent text.

**Table 4:** The distribution of NNC, HHH, and SC cultivars in seven genetic clusters.

**Subpopulation**	**Neighbor-joining cluster group**							**Total**
	**A**	**B**	**C**	**D**	**E**	**F**	**G**	
NNC	150	108	4	17				279
HHH		5	20	64	60	6		155
SC		7	8	2	12	35	62	126
Total	150	120	32	83	72	41	62	560

*NNC, HHH, and SC represent cultivars released in Northeast and Northwest China, Huang-Huai-Hai Valleys, and Southern China, respectively*.

### Genetic Differentiation of Modern Chinese Soybean Cultivars in Terms of Ancestor-Derived Cultivar Families

According to the progeny-pedigrees of the population-level core–terminal ancestors, those with the largest numbers of derived cultivars and high nuclear and cytoplasmic genetic contributions were recognized as major core–terminal ancestors, which along with its derived cultivars, were called a core-terminal ancestor-derived family. In this way, 11 major core-terminal ancestor-derived families were identified in the MCSCP. Their core-terminal ancestors were traced to CA-01 (Baimei), CA-02 (Jinyuan), CA-03 (Silihuang), and CA-05 (Duludou) from NNC, CA-15 (Binhaidabaihua), CA-16 (Tongshantianedan), CA-19 (Shouzhangdifangzhong) and CA-20 (Jimoyoudou) from HHH, CA-26 (Vegetable soybean population in Wuhan), CA-27 (Fengxiansuidaohuang) and CA-28 (51-83) from SC. These 11 major core–terminal ancestors out of the 604 terminal ancestors provided 100.65/560 = 17.97% nuclear genetic contribution and 177/560 = 31.6% cytoplasmic contribution to the MCSCP (Table 5).

**Table 5:** Frequency distribution among ecoregions and genetic parameters of the 11 major core-terminal ancestor-derived families.

**Code**	**Distribution of derived cultivars**				**NGC**	**CGC**	**Allele No**.				**π**	
	**NNC**	**HHH**	**SC**	**Total**			**Total**	**Per-locus**	**Per-cultivar**	**Max/Locus**	**Mean**	**Max**
CA-01	190	2	3	195	17.05	25	68,903	2.46	353.35	16	0.103	0.880
CA-02	233	47	16	296	14.32		81,909	2.92	276.72	21	0.114	0.906
CA-03	225	38	9	272	13.45	57	80,328	2.86	295.32	20	0.112	0.899
CA-05	150	50	20	220	11.42	20	81,790	2.91	371.77	21	0.120	0.918
CA-15	5	110	29	144	13.52	26	74,447	2.65	516.99	17	0.134	0.891
CA-16	2	101	24	127	6.29	1	72,803	2.59	573.25	16	0.134	0.882
CA-19	9	74	13	96	4.98	7	70,622	2.52	735.65	16	0.133	0.894
CA-20	13	83	17	113	4.59	9	73,412	2.62	649.66	18	0.135	0.918
CA-26			32	32	7.63	19	55,245	1.97	1726.41	11	0.140	0.881
CA-27		33	13	46	4.47	9	60,367	2.15	1312.33	13	0.133	0.901
CA-28		36	7	43	2.93	4	56,760	2.02	1320.00	12	0.129	0.904
Total	253(827)	137(574)	73(183)	463(1,584)	(100.65)	177	83,966	2.99	181.35	21	0.125	0.906

*Code: the code of the core terminal ancestors, and the corresponding name of the core terminal ancestors were shown in Supplementary Table 6. The derived cultivars may duplicate among the core-terminal ancestor-derived families, so the total cultivars were 463 while the total cultivar • time was 1,584 (in parentheses) and the same is for each ecoregion and for NGC (not for CGC). Per-cultivar means per-cultivar allele load calculated from the total allele number divided by the number of derived cultivars, which is a measure of the allele richness in a core-terminal ancestor-derived family*.

From the 11 major core-terminal ancestors, 463 cultivars were derived, and each cultivar involved one or more major core–terminal ancestors. Therefore, a total of 1,584 cultivar • times involved with the 11 major core-terminal ancestors. Among the 1,584 cultivar • times, 983 ones were from the four NNC major core-terminal ancestors, in which 798 cultivar • times for NNC cultivars, 137 cultivar • times for HHH cultivars and 48 cultivar • times for SC cultivars. While among the 1,584 cultivar • times, 480 cultivar • times were from the four HHH major core-terminal ancestors, in which 368 cultivar • times for HHH cultivars, 29 cultivar • times for NNC cultivars, and 83 cultivar • times for SC cultivars. Furthermore, among the 1,584 cultivar • times, 121 cultivar • times were from the three SC major core-terminal ancestors, in which 52 cultivar • times for SC cultivars, 69 cultivar • times for HHH cultivars, and none for NNC cultivars. Thus, there showed germplasm exchange among the three ecoregions regarding the 11 major core-terminal ancestor-derived families, the NNC germplasm and HHH germplasm penetrated to the other two regions, but no SC germplasm to NNC, especially CA-26 even not to HHH. In the 560 MCSCs, 56.24/560 = 10.04% nuclear and 102/560 = 18.21% cytoplasmic germplasm were from the four NNC major core-terminal ancestors, 29.38/560 = 5.25% nuclear and 43/560 = 7.68% cytoplasmic germplasm were from the four HHH major core-terminal ancestors, and only 15.03/560 = 2.68% nuclear and 32/560 = 5.71% cytoplasmic germplasm were from the three SC major core-terminal ancestors. It seems that both nuclear and cytoplasmic germplasm of the core-terminal ancestor-derived families might dominate in the MCSCP (Table 5).

As for the allele constitution, the total number of alleles in the 11 major ancestor-derived families ranged from 55,245 to 81,909, and the richness of NNC major ancestor-derived families (in an average of 78232.5) was more than those of HHH ones (in an average of 72,821), and in turn more than those of SC (in an average of 57457.3). The same tendency was for per-locus allele number (2.46–2.92, 2.52–2.65, and 1.97–2.15 for NNC, HHH, and SC major ancestor-derived families, respectively) and allele frequency dispersion (0.103–0.12, 0.133–0.135, and 0.129–0.14 for NNC, HHH, and SC major ancestor-derived families, respectively) but in the opposite situation for per-cultivar allele load (276.72–371.77, 516.99–735.65, and 1312.33–1726.41 for NNC, HHH, and SC major ancestor-derived families, respectively). That means the NNC major ancestor-derived families are richer in their allele sources and per-locus allele differentiation but are poorer in their per-cultivar allele load than that of the other two major ancestor-derived family groups (Table 5).

The results of neighbor-joining clustering showed that the 11 major ancestor-derived families were classified into three groups (Figure 4B). Group I is the most advanced cluster with the shortest distances to the root, which includes derived-families from CA-05, CA-02, CA-01, and CA-03, all the four major core-terminal ancestors were from NNC. Group II includes derived families from CA-15, CA-20, CA-16, and CA-19 (from HHH) CA-27, and CA-28 (from SC). This group included major core-terminal ancestors from both HHH and SC, indicating the relatively close relationship between the two sets of the ancestor-derived families. Group III includes the CA-26 derived family, which was the most primitive one with the longest distance to the root among the 11 families. CA-26 is a vegetable soybean population in SC.

### Features of COP and CGS in Evaluating the Genetic Similarity/Diversity Among the Released Cultivars

In studying the genetic relationship among released cultivars, COP was used at first based on parental pedigrees, while after the molecular marker technique developed, CGS was used extensively. COP represents the probability of the two cultivars carrying the same allele inherited from the same parent, i.e., identity by descent (IBD) (Hallauer and Miranda, 1981). In contrast, CGS represents the probability of the two cultivars carrying the same allele, regardless of the same parent, i.e., identity by state (IBS) (Hallauer and Miranda, 1981). A larger COP or CGS indicates a closer genetic relationship between the two cultivars. For convenience, all the calculated values of COP and CGS in the present study were multiplied by 100. The COP of the MCSCP was averaged to 1.04, not closely genetic-related among cultivars in the MCSCP, varying from 0 to 100, with a large variation from non-genetic relationship to full genetic relatedness (Table 6). Among the subpopulations, the COP values of NNC, HHH, and SC were averaged at 2.74, 2.11, and 1.15 respectively, while in NNC, it ranged from 0 to 70 and from 0 to 100 in HHH and SC. The COP of the most cultivar pairs concentrated in the groups of 0 and 0–10 (56.14 + 42.11% in the MCSCP, 20.90 + 74.01% in NNC, 29.37 + 67.52% in HHH, and 82.18 + 14.69% in SC), indicating NNC and HHH with more relatedness among cultivars than SC or more distant among cultivars in SC. In Table 6, the CGS of the MCSCP was averaged 87.23 in the MCSCs, varying from 74.62 to 98.73. Among the subpopulations, the CGS values were 89.35, 87.08, and 85.35 in NNC, HHH, and SC, ranging from 76.73 to 96.59, 81.02 to 98.73, and 79.48 to 96.18, respectively. In the MCSCP, the CGS of the most cultivar pairs concentrated in the groups of 80–90 and 90–100 (82.49 + 15.78% in MCSCP, 52.41 + 47.10% in NNC, 92.75 + 7.25% in HHH, and 98.41 + 1.35% in SC), indicating also NNC with more relatedness among cultivars than the latter two or more distant among cultivars in HHH and SC. Both COP and CGS confirmed that differentiation of genetic relatedness also happened among subpopulations due to geographic and agronomic differences. The shortened growth season caused a mono-/single-cropping system in NNC, an extended growth season caused a double-cropping system in HHH, and a long growth season caused multiple cropping systems in SC. Altogether happened the differentiation in requirements to cultivars and their parentage materials, therefore the genetic relatedness among cultivars.

**Table 6:** Frequency distribution of COP and CGS in NNC, HHH, and SC of the MCSCP.

**Population**	**COP** **set**												**Mean**
	**0**	**0–10**	**10–20**	**20–30**	**30–40**	**40–50**	**50–60**	**60–70**	**70–80**	**80–90**	**90–100**	**Total**	
NNC	8,104 (20.90)	28,702 (74.01)	1,365 (3.52)	442 (1.14)	113 (0.29)	37 (0.10)	17 (0.04)	1 (0.00)				38,781 (100)	2.74
HHH	3,505 (29.37)	8,059 (67.52)	176 (1.47)	150 (1.26)	16 (0.13)	16 (0.13)	10 (0.08)			1 (0.01)	2 (0.02)	11,935 (100)	2.11
SC	6,472 (82.18)	1,157 (14.69)	96 (1.22)	97 (1.23)	17 (0.22)	26 (0.33)		3 (0.04)	1 (0.01)	3 (0.04)	3 (0.04)	7,875 (100)	1.15
MCSCP	87,873 (56.14)	65,903 (42.11)	1,731 (1.11)	734 (0.47)	150 (0.10)	88 (0.06)	27 (0.02)	4 (0.00)	1 (0.00)	4 (0.00)	5 (0.00)	15,6520 (100)	1.04
**Population**	**CGS** **set**												**Mean**
	**0**	**0–10**	**10–20**	**20–30**	**30–40**	**40–50**	**50–60**	**60–70**	**70–80**	**80–90**	**90–100**	**Total**	
NNC									189 (0.49)	20,326 (52.41)	18,266 (47.10)	38,781 (100)	89.35
HHH										11,070 (92.75)	865 (7.25)	11,935 (100)	87.08
SC									19 (0.24)	7,750 (98.41)	106 (1.35)	7,875 (100)	85.35
MCSCP									2,703 (1.73)	129,116 (82.49)	24,701 (15.78)	156,520 (100)	87.23

*COP, coefficient of parentage, CGS, coefficient of genetic similarity*.

*In the “Population” column, NNC, HHH, and SC represent cultivars released in Northeast and Northwest China, Huang-Huai-Hai Valleys, and Southern China, respectively. MCSCP represents the modern Chinese soybean cultivar population*.

*The number of cultivar pairs is outside of the parentheses, while the percentage of frequency is in parentheses*.

*In the “Mean” column, the number was the mean value of COP or CGS of the population, which was multiplied by 100*.

The correlation between the COP and CGS matrices was *r* = 0.229, significant under the Mantel test in the MCSCP. Supplementary Table 8 showed the two-way table of COP and CGS frequency distribution. In the MCSCP, the most cultivar pairs concentrated in the four cells of COP groups of 0–0 and 0–2.5 by CGS groups of 84–89 and 89–94. The data indicated that the COP and CGS were correlated, but not closely, the two genetic relationship indicators have their own features. COP is characterized in that it is based on exact parental pedigrees, otherwise not possible to be calculated, especially, since the unknown relatedness between ancestors is not easy to be recognized (0 or 1 has to be assumed). In addition, it represents a theoretical genetic relationship useful in inheritance analysis, but not an exact one due to the fluctuation in segregation departing from the assumption of the equal genetic contribution of the parents to progenies. CGS is characterized that it is based on experimental data with full information from each cultivar/individual, especially, providing exact calculations coinciding with the real case, and that no assuming value (0 or 1) is required and easy in the algorithm, such as in GWAS procedures. Thus, in experimental genetics, CGS is more pragmatic than COP in studying the genetic relationship among/between cultivars.

## Discussion

### Pedigree Integrated With Genomic-Marker (SNPLDB) Analysis Advanced the Germplasm Study

In the study of released cultivars as germplasm sources, pedigree analysis has been a major approach. For a cultivar, there are pedigrees of its parental ancestors and pedigrees of its progenies, here we called parental pedigree and progeny pedigree, respectively. In the present study, in tracing the released cultivars' parental ancestors, the parental pedigree analysis was utilized while in tracing core-terminal ancestor-derived families, the progeny pedigree analysis was applied.

In soybean, parental pedigree analysis was the major procedure in studying the genetic basis of released cultivars before the molecular marker and genomic analysis. Delannay et al. (1983) and Gizlice et al. (1994) analyzed the pedigrees of North American cultivars released in different periods and traced their ancestors. Cheng and Hadley (1986) and Bernard (1988) analyzed the sources, pedigrees, and ancestors of cultivars released in the US and Canada in different periods. Hiromoto (1986), Zhou et al. (2000), Bhardwaj et al. (2002), and Wysmierski and Vello (2013) analyzed the pedigree of Brazilian, Japanese, Indian, and Brazilian soybean cultivars, then summarized the main ancestors or core ancestors in a certain period, respectively. These were of great significance to germplasm exchange among different regions in broadening the genetic basis of cultivars. In the present study, 604 terminal ancestors were summarized from the 560 cultivars, and 41 core-terminal ancestors were extracted from them as the representatives of important terminal ancestors in the Chinese soybean germplasm population, and then the germplasm sources of the MCSCP were clarified from the perspectives of the parental pedigrees. In comparison to Xiong et al. (2007), the 41 presently nominated population-level core-terminal ancestors in 2006–2015 were included in those during 1923–2005, but their frequencies changed. That means the germplasm exchange among ecoregions during the recent period (2006–2015) did not shack the basic genetic background established in 1923–2005. In other words, the MCSCs have inherited the superior genes-alleles from those released in about a century, while the gene exchange through introducing new parental materials may have caused some changes due to the differences in inter-regional breeding activities but not very much (Figure 3). In the MCSCP, the core-terminal ancestors shared between HHH and SC increased by 5.12%, while that between HHH and NNC decreased by 15.93%. It indicates that the germplasm infiltration from HHH to SC is more common than from HHH to NNC, therefore, the germplasm exchange among subpopulations has been being appealed during recent years. On the other hand, the progeny pedigree analysis was based in fact on parental analysis of the derived cultivars, i.e., the 41 core-terminal ancestor and 11 major core-terminal ancestor-derived families were based on the parental pedigree analysis of 560 and 463 cultivars, respectively. From the analysis, the most important germplasm sources in the MCSCP were summarized.

Genetic markers helped to characterize the genetic constitution, the genetic difference among cultivars, and genetic differentiation among the subpopulations using genetic richness, SPA, SDA, allele frequency dispersion, and genetic similarity/diversity as indicated in the results. Using the marker-assisted analysis, the multiple germplasm sources of the geographic subpopulations were found through clustering analysis. Especially, the genomic markers SNPLDBs with multiple alleles were used which can cover the whole genome and provide relatively thorough genetic information. Therefore, the pedigree analysis integrated with genomic-maker SNPLDB analysis advanced the present study on released cultivars as growing germplasm sources.

### Geographic and Breeding-Effort Factors Involved in the Differentiation of the MCSCP and Implications for Breeding for Soybeans

In the results section, the differentiation of the MCSCP was analyzed from three aspects, i.e., geographic differentiation, genetic structure differentiation, and genetic clustering differentiation due to ancestor changes, which involved geographic factor and breeding-effort/parentage factor. At the early stage of the population differentiation, the geographic factors determined the differentiation direction for getting soybeans adaptive to local environments, including geographic photo-thermal and sowing seasonal conditions (cropping system). Later on, the exchange of parental materials caused the outside germplasm introduced into the local region. As indicated above, although the soybean production history was very long in China, the scientific breeding history was only about one century, while the germplasm exchange history was now at its early stage.

In comparison, the American soybean cultivars, which were developed based on mainly the introductions of Northeast China germplasm during a recent couple of centuries in their scientific breeding plans, but the American cultivars have their yield potential exceeding their Chinese ancestors. Liu et al. (2020) reported that in the global clustering of the 13 geographic soybean subpopulations, those of the north and south Americas were clustered with those of Northeast China in the same group, while the latter was not with the Asian group. Now the American soybeans have covered all 13 MGs, almost the same as the Asian group. The Asian group took a long history to get soybean cultivars developed under a relatively self-sufficient manner in using germplasm while the American group took a very short period to get soybean cultivars developed under a broad exchanging manner in using germplasm. This is an obvious example of the global differentiation of soybeans due to the breeding-effort/parentage factor in addition to the geographic factor.

Gizlice et al. (1994) found that 28 ancestors and seven first progenies contributed 95.97% of the genes found in public cultivars released between 1947 and 1988 in North America. In these 35 cultivars, four ancestors (Mandarin, Richland, A.K., and Dunfield) and one derived-progeny (Lincoln) contributed 46.73% of the genes. The five accessions also existed in the 41 core-terminal ancestors of 2006–2015 and in the 113 core-terminal ancestors of 1923–2005 in the Chinese soybean population (Xiong et al., 2007), providing 5.51 and 5.7% germplasm contribution, respectively. This indicates that the germplasm from outside of China should be introduced back and utilized in soybean improvement in the three eco-regions because the outside germplasm has been improved by foreign breeders even though its source was initially based on the Chinese introductions.

In essence, breeding is a process of introducing and accumulating superior genes/alleles of target traits and eliminating inefficient genes/alleles from different parents. Wild soybeans and landraces generally contain excellent or special trait genes, but their comprehensive genetic background is poor and unsuitable to be directly used in production, so they are only suitable as donor parents. Released cultivars are an important and the most widely used type of soybean germplasm resource, especially the recently released cultivars, which have been improved for several breeding cycles and can meet the production demands at the present stage. Therefore, released cultivars are important receptor materials to receive complimentary superior genes/alleles from donors for further improvement in the future.

The above results showed that the germplasm sources of the three regions are relatively independent, except for some small amount of exchange and utilization happened. For further soybean improvement in China, the germplasm exchange among ecoregions should be enhanced and at the same time, the introduction of excellent germplasm resources from abroad should be emphasized also for adding new alleles or allele-recombinants to generate new genetic types, broadening the genetic base of the local population and enriching the genetic diversity of future cultivars.

## Conclusions

The modern released cultivar population is a genetic deposition of historical accumulation of breeding effort or superior genes-alleles. The MCSCP, composed of 560 released cultivars (279 in NNC, 155 in HHH, and 126 in SC), covered a wide range of geo-seasonal ecotypes in terms of maturity groups (MG 000–MG IX). Its germplasm sources were traced to 718 immediate parents, 604 terminal ancestors, and 41 core-terminal ancestors through parental-pedigree analysis of the released cultivars. Among the three eco-subpopulations, the germplasm sources were different from each other with a part shared by two or three eco-subpopulations (2.65% immediate parents, 19.87% terminal-ancestors), while 87.81% core-terminal ancestors shared by two or three eco-subpopulations. The genomic marker analysis further indicated the MCSCP differentiation among the three subpopulations, NNC with more genetic richness (74,386 alleles vs. 72,894 and 72,843 alleles), more SPA, and SDA than the others (4,603 and 6,729 vs. 560 and 4,198, and 2,374 and 6,061 alleles, respectively). In evaluating genetic relationship/differentiation, CGS is more pragmatic than COP. The CGS results confirmed NNC was less distant from HHH (CGS 87.32%) in comparison to SC (CGS 86.12%) and less distant than to that between HHH and SC (CGS 85.66%). The genetic clustering of the MCSCP further supported the genetic differentiation among the three subpopulations but was partially caused by the interchange of genetic sources. Combining the parental-pedigree with genomic marker analysis, the major core-terminal ancestor-derived families are also characterized by eco-regional differentiation. All the results showed that the germplasm sources of the three eco-subpopulations are relatively self-sufficient, germplasm exchange only at an early stage, for further improvement, domestic and international germplasm exchange is seriously required.

## Data Availability Statement

The original contributions presented in the study are included in the article/Supplementary Material; the raw data supporting the conclusions of this article will be made available by the authors, without undue reservation.

## Author Contributions

CLi, JZ, and JG conceived the study and drafted, revised, and finalized the manuscript. CLi, JZ, CLiu, XZ, and JC collected the cultivars with their pedigrees. CLi, CLiu, XZ, JC, and WW performed the experiment. CLi, WW, YP, JH, and FL performed data analysis. All authors approved the submitted manuscript.

## Funding

This work was supported by the National Key Research Development Program of China (2021YFF1001204), the National Natural Science Foundation of China (32072082), the Program of Jiangsu Province (JBGS-2021-014), the MOE 111 Project (B08025), the China Agriculture Research System of MOF and MARA (CARS-04), the Primary Research & Development Plan of Jiangsu Province (BE2021358), the Jiangsu JCIC-MCP, the Cyrus Tang Innovation Center for Seed Industry (CTIC-SI201902) and the Guidance Foundation of Sanya Institute of Nanjing Agricultural University (NAUSY-ZZ02, NAUSY-MS05).

## Conflict of Interest

The authors declare that the research was conducted in the absence of any commercial or financial relationships that could be construed as a potential conflict of interest.

## Publisher's Note

All claims expressed in this article are solely those of the authors and do not necessarily represent those of their affiliated organizations, or those of the publisher, the editors and the reviewers. Any product that may be evaluated in this article, or claim that may be made by its manufacturer, is not guaranteed or endorsed by the publisher.
