# Supplementary material for: Germplasm Sources, Genetic Richness, and Population Differentiation of Modern Chinese Soybean Cultivars Based on Pedigree Integrated With Genomic-Marker Analysis
Source: Front Plant Sci. 2022 Jul 11;13:945839. doi: 10.3389/fpls.2022.945839 (PMC9309878; doi:10.3389/fpls.2022.945839)
Supplement: Supplementary file 1 [file Data_Sheet_1.docx]

Supplementary Materials

# Supplementary Figures


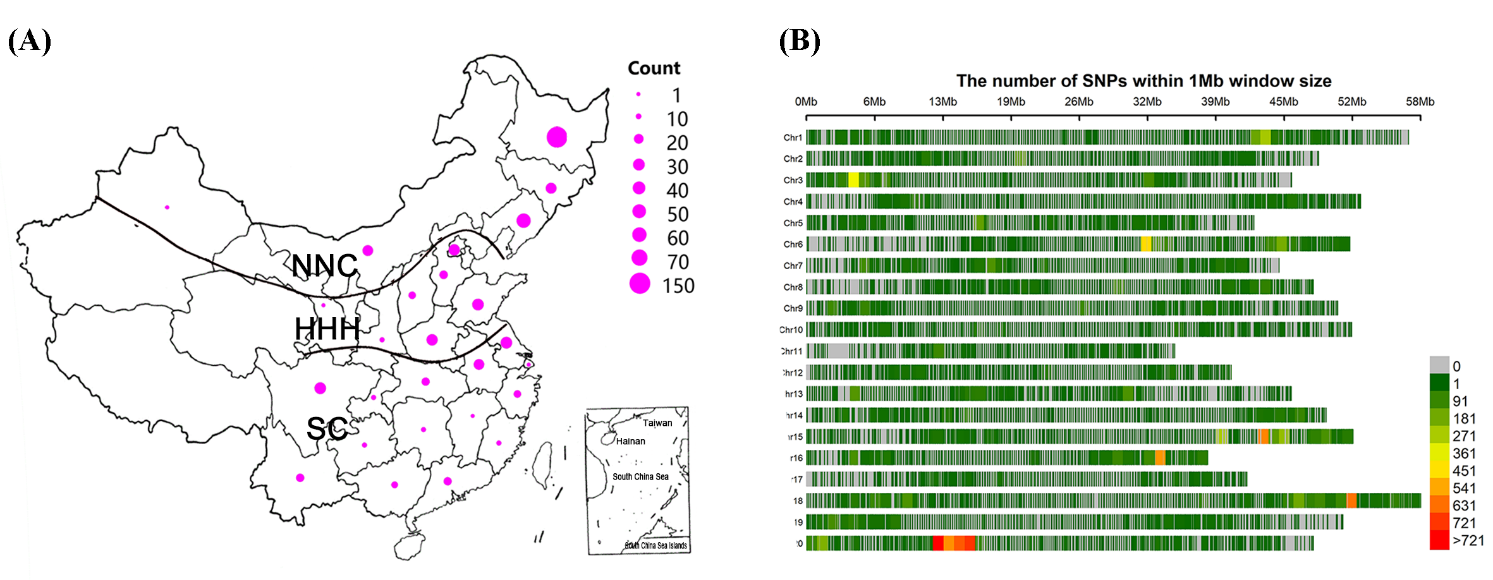


## Supplementary Figure 1. Distribution of the MCSCs in three ecoregions and distribution of SNPs on chromosomes of the MCSCP.

MCSCs and MCSCP represent the modern Chinese soybean cultivars and the modern Chinese soybean cultivar population. (A) Geographical distribution of the 560 MCSCs. The red dots indicate the number of cultivars from the corresponding provinces. NNC, HHH and SC represent Northeast and Northwest China, Huang-Huai-Hai Valleys and Southern China, respectively. (B) Distribution of SNPs on chromosomes the MCSCP.


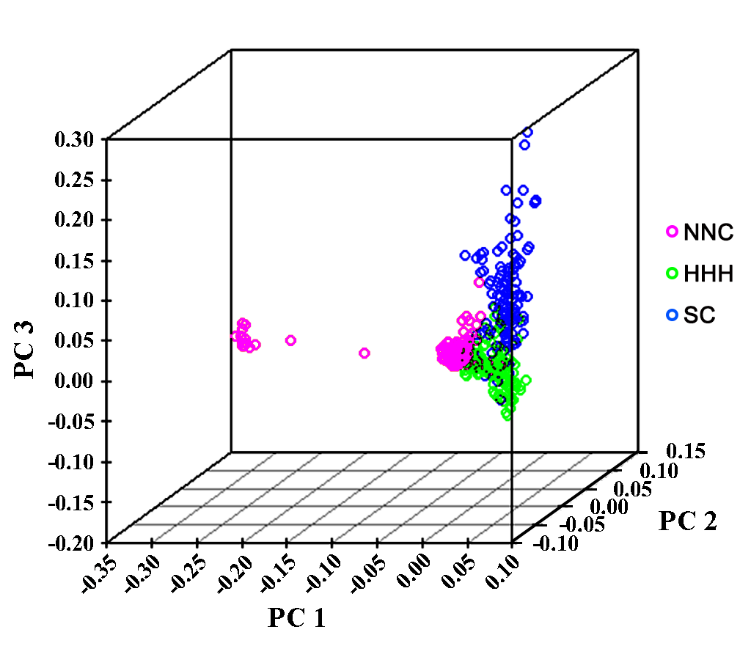


## Supplementary Figure 2. Principal component analysis of the MCSCP.

NNC, HHH and SC represent cultivars released in Northeast and Northwest China, Huang-Huai-Hai Valleys and Southern China, respectively. The first, second and third components explain 87.69%, 0.61% and 0.34% of the total variation.

# Supplementary Tables

## Supplementary Table 1. The source-provinces of the MCSCP.

| **Source province** | **NNC** | **HHH** | **SC** | **Total** |
| --- | --- | --- | --- | --- |
| Heilongjiang | 156 (26) |  |  | 156 (26) |
| Jilin | 25 (4) |  |  | 25 (4) |
| Liaoning | 65 (6) |  |  | 65 (6) |
| Neimenggu | 24 (1) |  |  | 24 (1) |
| Xinjiang | 1 |  |  | 1 |
| Anhui |  | 24 (6) |  | 24 (6) |
| Beijing | 4 (1) | 23 (8) |  | 27 (9) |
| Gansu |  | 1 |  | 1 |
| Hebei | 4 | 21 (7) | 1 (1) | 26 (8) |
| Henan |  | 25 (7) | 4 (4) | 29 (11) |
| Jiangsu |  | 12 (4) | 23 (5) | 35 (9) |
| Shandong |  | 35 (11) |  | 35 (11) |
| Shānxi |  | 10 (5) |  | 10 (5) |
| Shǎnxi |  | 4 |  | 4 |
| Fujian |  |  | 4 | 4 |
| Guangdong |  |  | 11 (8) | 11 (8) |
| Guangxi |  |  | 12 (1) | 12 (1) |
| Guizhou |  |  | 4 (2) | 4 (2) |
| Hubei |  |  | 11 (7) | 11 (7) |
| Hunan |  |  | 4 (1) | 4 (1) |
| Jiangxi |  |  | 1 | 1 |
| Shanghai |  |  | 2 (1) | 2 (1) |
| Sichuan |  |  | 32 (2) | 32 (2) |
| Yunnan |  |  | 3 (3) | 3 (3) |
| Zhejiang |  |  | 10 (1) | 10 (1) |
| Chongqing |  |  | 4 | 4 |
| Total | 279 (38) | 155 (48) | 126 (36) | 560 (122) |

NNC, HHH and SC represent Northeast and Northwest China, Huang-Huai-Hai Valleys and Southern China, respectively. MCSCP represents the modern Chinese soybean cultivar population.

Outside of the parentheses is the sum of state-authorized and province-authorized cultivars, and in parentheses, is the state-authorized cultivars. A cell without parentheses means no state-authorized cultivar released.

## Supplementary Table 2. The passport data for the 560 cultivars in MCSCP.

| **Code** | **Cultivar** | **Source province** | **Maturity Group** | **Flower color** | **Seed coat color** |
| --- | --- | --- | --- | --- | --- |
| MC001 | Beidou 10 hao | Heilongjiang | 0 | purple | yellow |
| MC002 | Beidou 18 | Heilongjiang | 0 | purple | yellow |
| MC003 | Beidou 19 | Heilongjiang | 0 | purple | yellow |
| MC004 | Beidou 20 | Heilongjiang | 00 | purple | yellow |
| MC005 | Beidou 21 | Heilongjiang | 0 | purple | yellow |
| MC006 | Beidou 22 | Heilongjiang | 00 | purple | yellow |
| MC007 | Beidou 23 | Heilongjiang | 00 | purple | yellow |
| MC008 | Beidou 26 | Heilongjiang | 000 | purple | yellow |
| MC009 | Beidou 35 | Heilongjiang | 0 | purple | yellow |
| MC010 | Beidou 36 | Heilongjiang | 000 | purple | yellow |
| MC011 | Beidou 37 | Heilongjiang | 0 | white | yellow |
| MC012 | Beidou 40 | Heilongjiang | I | purple | yellow |
| MC013 | Beidou 42 | Heilongjiang | 00 | purple | yellow |
| MC014 | Beidou 43 | Heilongjiang | 000 | purple | yellow |
| MC015 | Beidou 51 | Heilongjiang | 000 | purple | yellow |
| MC016 | Beidou 53 | Heilongjiang | 00 | purple | yellow |
| MC017 | Beidou 54 | Heilongjiang | 0 | white | yellow |
| MC018 | Beidou 7 hao | Heilongjiang | 00 | purple | yellow |
| MC019 | Beidou 9 hao | Heilongjiang | 0 | purple | yellow |
| MC020 | Dongnong 49 | Heilongjiang | 00 | white | yellow |
| MC021 | Dongnong 50 | Heilongjiang | 0 | white | yellow |
| MC022 | Dongnong 51 | Heilongjiang | 0 | white | yellow |
| MC023 | Dongnong 52 | Heilongjiang | I | purple | yellow |
| MC024 | Dongnong 53 | Heilongjiang | 0 | purple | yellow |
| MC025 | Dongnong 54 | Heilongjiang | I | purple | yellow |
| MC026 | Dongnong 55 | Heilongjiang | I | purple | yellow |
| MC027 | Dongnong 56 | Heilongjiang | 0 | purple | yellow |
| MC028 | Dongnong 57 | Heilongjiang | I | white | green |
| MC029 | Dongnong 58 | Heilongjiang | 000 | purple | yellow |
| MC030 | Dongnong 59 | Heilongjiang | 0 | purple | yellow |
| MC031 | Dongnong 60 | Heilongjiang | 0 | purple | yellow |
| MC032 | Dongnong 61 | Heilongjiang | I | purple | yellow |
| MC033 | Dongnong 62 | Heilongjiang | I | purple | yellow |
| MC034 | Dongnong 63 | Heilongjiang | 00 | purple | yellow |
| MC035 | Dongnong 65 | Heilongjiang | 0 | purple | yellow |
| MC036 | Dongsheng 2 hao | Heilongjiang | 0 | purple | yellow |
| MC037 | Dongsheng 77 | Heilongjiang | 00 | purple | yellow |
| MC038 | Fengdou 3 hao | Heilongjiang | 00 | white | yellow |
| MC039 | Fengshou 25 | Heilongjiang | 0 | white | yellow |
| MC040 | Fengshou 26 | Heilongjiang | 0 | purple | yellow |
| MC041 | Fengshou 27 | Heilongjiang | 0 | purple | yellow |
| MC042 | Guangshilvdadou 1 hao | Heilongjiang | 0 | white | green |
| MC043 | Guangxingheidadou 1 hao | Heilongjiang | 00 | white | black |
| MC044 | Hefeng 50 | Heilongjiang | 0 | purple | yellow |
| MC045 | Hefeng 51 | Heilongjiang | 0 | purple | yellow |
| MC046 | Hefeng 52 | Heilongjiang | 0 | white | yellow |
| MC047 | Hefeng 54 | Heilongjiang | 0 | white | yellow |
| MC048 | Hefeng 55 | Heilongjiang | 0 | purple | yellow |
| MC049 | Hefeng 56 | Heilongjiang | 0 | purple | yellow |
| MC050 | Henong 58 | Heilongjiang | 0 | white | yellow |
| MC051 | Henong 60 | Heilongjiang | 0 | white | yellow |
| MC052 | Henong 63 | Heilongjiang | 0 | purple | yellow |
| MC053 | Henong 64 | Heilongjiang | 0 | white | yellow |
| MC054 | Henong 65 | Heilongjiang | 0 | white | yellow |
| MC055 | Henong 66 | Heilongjiang | 0 | purple | yellow |
| MC056 | Henong 67 | Heilongjiang | 0 | purple | yellow |
| MC057 | Henong 68 | Heilongjiang | 0 | purple | yellow |
| MC058 | Henong 69 | Heilongjiang | 0 | white | yellow |
| MC059 | Henong 70 | Heilongjiang | 0 | purple | yellow |
| MC060 | Henong 71 | Heilongjiang | 0 | purple | yellow |
| MC061 | Henong 75 | Heilongjiang | 0 | purple | yellow |
| MC062 | Henong 76 | Heilongjiang | 0 | purple | yellow |
| MC063 | Heihe 39 | Heilongjiang | 00 | purple | yellow |
| MC064 | Heihe 43 | Heilongjiang | 00 | purple | yellow |
| MC065 | Heihe 44 | Heilongjiang | 000 | purple | yellow |
| MC066 | Heihe 45 | Heilongjiang | 00 | purple | yellow |
| MC067 | Heihe 46 | Heilongjiang | 0 | purple | yellow |
| MC068 | Heihe 48 | Heilongjiang | 00 | purple | yellow |
| MC069 | Heihe 49 | Heilongjiang | 000 | white | yellow |
| MC070 | Heihe 50 | Heilongjiang | 00 | purple | yellow |
| MC071 | Heihe 52 | Heilongjiang | 00 | purple | yellow |
| MC072 | Heihe 53 | Heilongjiang | 0 | white | yellow |
| MC073 | Heike 56 | Heilongjiang | 000 | white | yellow |
| MC074 | Heinong 50 | Heilongjiang | I | purple | yellow |
| MC075 | Heinong 51 | Heilongjiang | I | white | yellow |
| MC076 | Heinong 52 | Heilongjiang | I | purple | yellow |
| MC077 | Heinong 53 | Heilongjiang | I | purple | yellow |
| MC078 | Heinong 55 | Heilongjiang | I | white | yellow |
| MC079 | Heinong 59 | Heilongjiang | 0 | white | yellow |
| MC080 | Heinong 60 | Heilongjiang | 0 | white | yellow |
| MC081 | Heinong 61 | Heilongjiang | I | purple | yellow |
| MC082 | Heinong 66 | Heilongjiang | 0 | white | yellow |
| MC083 | Heinong 67 | Heilongjiang | I | purple | yellow |
| MC084 | Heinong 68 | Heilongjiang | I | white | yellow |
| MC085 | Heinong 69 | Heilongjiang | I | purple | yellow |
| MC086 | Heinong 70 | Heilongjiang | 0 | white | yellow |
| MC087 | Huacaidou 1 hao | Heilongjiang | 00 | purple | yellow |
| MC088 | Huajiang 2 hao | Heilongjiang | 000 | purple | yellow |
| MC089 | Huajiang 4 hao | Heilongjiang | 0 | purple | yellow |
| MC090 | Jinyuan 55 | Heilongjiang | 00 | white | yellow |
| MC091 | Kangxianchong 10 hao | Heilongjiang | I | white | yellow |
| MC092 | Kangxianchong 11 | Heilongjiang | I | purple | yellow |
| MC093 | Kangxianchong 12 | Heilongjiang | I | purple | yellow |
| MC094 | Kangxianchong 6 hao | Heilongjiang | I | white | yellow |
| MC095 | Kangxianchong 7 hao | Heilongjiang | I | white | yellow |
| MC096 | Kangxianchong 8 hao | Heilongjiang | I | white | yellow |
| MC097 | Kangxianchong 9 hao | Heilongjiang | I | white | yellow |
| MC098 | Kedou 28 | Heilongjiang | 0 | white | yellow |
| MC099 | Keshan 1 hao | Heilongjiang | 00 | purple | yellow |
| MC100 | Kenbaoxiaolidou 1 hao | Heilongjiang | 0 | white | yellow |
| MC101 | Kendou 18 | Heilongjiang | 0 | purple | yellow |
| MC102 | Kendou 25 | Heilongjiang | 0 | white | yellow |
| MC103 | Kendou 30 | Heilongjiang | 0 | white | yellow |
| MC104 | Kendou 33 | Heilongjiang | 0 | white | yellow |
| MC105 | Kendou 36 | Heilongjiang | 0 | white | yellow |
| MC106 | Kendou 38 | Heilongjiang | 0 | white | yellow |
| MC107 | Kendou 39 | Heilongjiang | 0 | purple | yellow |
| MC108 | Kendou 40 | Heilongjiang | 0 | purple | yellow |
| MC109 | Kendou 43 | Heilongjiang | 0 | purple | yellow |
| MC110 | Kenfeng 16 | Heilongjiang | 0 | white | yellow |
| MC111 | Kenfeng 17 | Heilongjiang | 0 | purple | yellow |
| MC112 | Kenfeng 22 | Heilongjiang | 0 | purple | yellow |
| MC113 | Kenfeng 23 | Heilongjiang | I | purple | yellow |
| MC114 | Kennong 23 | Heilongjiang | I | purple | yellow |
| MC115 | Kennong 26 | Heilongjiang | 0 | white | yellow |
| MC116 | Kennong 28 | Heilongjiang | 0 | purple | yellow |
| MC117 | Kennong 29 | Heilongjiang | 0 | purple | yellow |
| MC118 | Kennong 30 | Heilongjiang | 0 | white | yellow |
| MC119 | Kennong 40 | Heilongjiang | 0 | purple | yellow |
| MC120 | Mudou 8 hao | Heilongjiang | I | purple | yellow |
| MC121 | Mudou 9 hao | Heilongjiang | I | purple | yellow |
| MC122 | Mufeng 7 hao | Heilongjiang | I | white | yellow |
| MC123 | Mushi 1 hao | Heilongjiang | 0 | white | yellow |
| MC124 | Nenao 1 hao | Heilongjiang | 00 | white | yellow |
| MC125 | Nenao 2 hao | Heilongjiang | 000 | purple | yellow |
| MC126 | Nenao 3 hao | Heilongjiang | 000 | white | yellow |
| MC127 | Nenao 4 hao | Heilongjiang | 00 | white | yellow |
| MC128 | Nenfeng 19 | Heilongjiang | 0 | white | yellow |
| MC129 | Pengcheng 158 | Heilongjiang | I | white | yellow |
| MC130 | Qinong 1 hao | Heilongjiang | 0 | white | yellow |
| MC131 | Qinong 2 hao | Heilongjiang | I | white | yellow |
| MC132 | Qingdou 13 | Heilongjiang | I | purple | yellow |
| MC133 | Suinong 23 | Heilongjiang | 0 | purple | yellow |
| MC134 | Suinong 24 | Heilongjiang | 0 | purple | yellow |
| MC135 | Suinong 25 | Heilongjiang | I | purple | yellow |
| MC136 | Suinong 26 | Heilongjiang | I | purple | yellow |
| MC137 | Suinong 27 | Heilongjiang | 0 | purple | yellow |
| MC138 | Suinong 28 | Heilongjiang | I | purple | yellow |
| MC139 | Suinong 29 | Heilongjiang | I | white | yellow |
| MC140 | Suinong 30 | Heilongjiang | 0 | purple | yellow |
| MC141 | Suinong 31 | Heilongjiang | I | purple | yellow |
| MC142 | Suinong 32 | Heilongjiang | 0 | purple | yellow |
| MC143 | Suinong 33 | Heilongjiang | I | purple | yellow |
| MC144 | Suinong 34 | Heilongjiang | 0 | white | yellow |
| MC145 | Suinong 35 | Heilongjiang | 0 | white | yellow |
| MC146 | Suinong 36 | Heilongjiang | I | white | yellow |
| MC147 | Suinong 37 | Heilongjiang | 0 | white | yellow |
| MC148 | Suinong 38 | Heilongjiang | I | white | yellow |
| MC149 | Suinong 39 | Heilongjiang | I | purple | yellow |
| MC150 | Suinong 41 | Heilongjiang | I | purple | yellow |
| MC151 | Suizhongzuo 40 | Heilongjiang | I | purple | yellow |
| MC152 | Xingnong 1 hao | Heilongjiang | I | white | yellow |
| MC153 | Xingnong 2 hao | Heilongjiang | 0 | white | yellow |
| MC154 | Xingnong 3 hao | Heilongjiang | I | white | yellow |
| MC155 | Xingnong 4 hao | Heilongjiang | 0 | purple | yellow |
| MC156 | Xingnonglvxiaolidou | Heilongjiang | 0 | purple | green |
| MC157 | Jidadou 1 hao | Jilin | I | purple | yellow |
| MC158 | Jidadou 2 hao | Jilin | I | white | yellow |
| MC159 | Jidadou 3 hao | Jilin | 0 | white | yellow |
| MC160 | Jidadou 5 hao | Jilin | 0 | purple | yellow |
| MC161 | Jinong 18 | Jilin | I | purple | yellow |
| MC162 | Jinong 20 | Jilin | I | white | yellow |
| MC163 | Jinong 22 | Jilin | III | purple | yellow |
| MC164 | Jinong 27 | Jilin | 0 | white | yellow |
| MC165 | Jinong 28 | Jilin | 0 | purple | yellow |
| MC166 | Jinong 29 | Jilin | I | white | yellow |
| MC167 | Jinong 31 | Jilin | I | purple | yellow |
| MC168 | Jinong 32 | Jilin | I | white | yellow |
| MC169 | Jinong 33 | Jilin | I | purple | yellow |
| MC170 | Jinong 34 | Jilin | 0 | purple | yellow |
| MC171 | Jinong 35 | Jilin | 0 | white | yellow |
| MC172 | Jinong 37 | Jilin | I | purple | yellow |
| MC173 | Jinong 38 | Jilin | 0 | white | yellow |
| MC174 | Jiunong 35 | Jilin | 0 | white | yellow |
| MC175 | Jiunong 36 | Jilin | II | purple | yellow |
| MC176 | Jiunong 39 | Jilin | III | purple | yellow |
| MC177 | Jiunong 40 | Jilin | 0 | purple | yellow |
| MC178 | Tongnong 15 | Jilin | 0 | purple | yellow |
| MC179 | Tongnong 943 | Jilin | I | white | yellow |
| MC180 | Yanyu 1 hao | Jilin | 0 | white | yellow |
| MC181 | Yanyu 2 hao | Jilin | 0 | white | yellow |
| MC182 | Dandou 13 | Liaoning | III | white | yellow |
| MC183 | Dandou 14 | Liaoning | III | white | yellow |
| MC184 | Dandou 15 | Liaoning | III | white | yellow |
| MC185 | Dandou 16 | Liaoning | III | purple | yellow |
| MC186 | Dandou 17 | Liaoning | III | purple | yellow |
| MC187 | Liaodou 18 | Liaoning | III | white | yellow |
| MC188 | Liaodou 23 | Liaoning | III | purple | yellow |
| MC189 | Liaodou 24 | Liaoning | III | purple | yellow |
| MC190 | Liaodou 25 | Liaoning | I | purple | yellow |
| MC191 | Liaodou 26 | Liaoning | III | purple | yellow |
| MC192 | Liaodou 28 | Liaoning | I | white | yellow |
| MC193 | Liaodou 29 | Liaoning | I | white | yellow |
| MC194 | Liaodou 30 | Liaoning | I | purple | yellow |
| MC195 | Liaodou 31 | Liaoning | III | white | yellow |
| MC196 | Liaodou 32 | Liaoning | II | purple | yellow |
| MC197 | Liaodou 33 | Liaoning | III | purple | yellow |
| MC198 | Liaodou 34 | Liaoning | IV | purple | yellow |
| MC199 | Liaodou 35 | Liaoning | III | purple | yellow |
| MC200 | Liaodou 36 | Liaoning | III | purple | yellow |
| MC201 | Liaodou 37 | Liaoning | I | purple | yellow |
| MC202 | Liaodou 38 | Liaoning | I | purple | yellow |
| MC203 | Liaodou 39 | Liaoning | I | white | yellow |
| MC204 | Liaodou 40 | Liaoning | I | purple | yellow |
| MC205 | Liaodou 41 | Liaoning | I | purple | yellow |
| MC206 | Liaodou 42 | Liaoning | I | white | yellow |
| MC207 | Liaodou 43 | Liaoning | I | purple | yellow |
| MC208 | Liaodou 44 | Liaoning | I | white | yellow |
| MC209 | Liaodou 45 | Liaoning | I | purple | yellow |
| MC210 | Shennongdou 19 | Liaoning | 0 | purple | yellow |
| MC211 | Shennongdou 20 | Liaoning | I | white | yellow |
| MC212 | Shennongdou 21 | Liaoning | 0 | purple | yellow |
| MC213 | Tiedou 39 | Liaoning | II | purple | yellow |
| MC214 | Tiedou 40 | Liaoning | IV | purple | yellow |
| MC215 | Tiedou 42 | Liaoning | I | purple | yellow |
| MC216 | Tiedou 43 | Liaoning | II | purple | yellow |
| MC217 | Tiedou 45 | Liaoning | IV | purple | yellow |
| MC218 | Tiedou 46 | Liaoning | III | purple | yellow |
| MC219 | Tiedou 47 | Liaoning | III | purple | yellow |
| MC220 | Tiedou 48 | Liaoning | IV | purple | yellow |
| MC221 | Tiedou 49 | Liaoning | III | purple | yellow |
| MC222 | Tiedou 50 | Liaoning | III | white | yellow |
| MC223 | Tiedou 51 | Liaoning | III | white | yellow |
| MC224 | Tiedou 53 | Liaoning | IV | purple | yellow |
| MC225 | Tiedou 54 | Liaoning | III | purple | yellow |
| MC226 | Tiedou 55 | Liaoning | III | purple | yellow |
| MC227 | Tiedou 56 | Liaoning | IV | purple | yellow |
| MC228 | Tiedou 58 | Liaoning | II | purple | yellow |
| MC229 | Tiedou 59 | Liaoning | IV | white | yellow |
| MC230 | Tiedou 60 | Liaoning | III | purple | yellow |
| MC231 | Tiedou 62 | Liaoning | III | purple | yellow |
| MC232 | Tiedou 63 | Liaoning | IV | purple | yellow |
| MC233 | Tiedou 64 | Liaoning | II | white | yellow |
| MC234 | Tiedou 65 | Liaoning | I | white | yellow |
| MC235 | Tiedou 66 | Liaoning | II | purple | yellow |
| MC236 | Tiedou 67 | Liaoning | II | white | yellow |
| MC237 | Tiedou 68 | Liaoning | I | purple | yellow |
| MC238 | Tiedou 70 | Liaoning | II | white | yellow |
| MC239 | Tiedou 71 | Liaoning | II | purple | yellow |
| MC240 | Tiedou 72 | Liaoning | II | white | yellow |
| MC241 | Tiedou 74 | Liaoning | I | purple | yellow |
| MC242 | Tiedou 75 | Liaoning | I | purple | yellow |
| MC243 | Tiedou 76 | Liaoning | I | purple | yellow |
| MC244 | Tiedou 77 | Liaoning | II | purple | yellow |
| MC245 | Tiedou 78 | Liaoning | II | purple | yellow |
| MC246 | Xiuyudou 1 hao | Liaoning | II | white | yellow |
| MC247 | Chidou 3 hao | Neimenggu | II | white | yellow |
| MC248 | Chidou 4 hao | Neimenggu | I | white | yellow |
| MC249 | Dengke 10 hao | Neimenggu | 00 | white | yellow |
| MC250 | Dengke 1 hao | Neimenggu | 0 | purple | yellow |
| MC251 | Dengke 3 hao | Neimenggu | 00 | purple | yellow |
| MC252 | Dengke 4 hao | Neimenggu | 0 | white | yellow |
| MC253 | Dengke 5 hao | Neimenggu | I | purple | yellow |
| MC254 | Dengke 6 hao | Neimenggu | 0 | purple | yellow |
| MC255 | Dengke 7 hao | Neimenggu | 00 | purple | yellow |
| MC256 | Dengke 8 hao | Neimenggu | 00 | purple | yellow |
| MC257 | Mengdou 19 | Neimenggu | 000 | purple | yellow |
| MC258 | Mengdou 21 | Neimenggu | 0 | white | yellow |
| MC259 | Mengdou 26 | Neimenggu | 00 | purple | yellow |
| MC260 | Mengdou 28 | Neimenggu | 0 | white | yellow |
| MC261 | Mengdou 30 | Neimenggu | 0 | white | yellow |
| MC262 | Mengdou 31 | Neimenggu | 000 | white | yellow |
| MC263 | Mengdou 32 | Neimenggu | 00 | purple | yellow |
| MC264 | Mengdou 33 | Neimenggu | 0 | white | yellow |
| MC265 | Mengdou 34 | Neimenggu | 0 | purple | yellow |
| MC266 | Mengdou 35 | Neimenggu | 0 | white | yellow |
| MC267 | Mengdou 36 | Neimenggu | 00 | purple | yellow |
| MC268 | Mengdou 37 | Neimenggu | 000 | white | yellow |
| MC269 | Mengdou 38 | Neimenggu | 00 | white | yellow |
| MC270 | Xingdou 5 hao | Neimenggu | I | purple | yellow |
| MC271 | Xindadou 27 | Xinjiang | 00 | white | yellow |
| MC272 | Anyi 13 | Anhui | II | purple | yellow |
| MC273 | Fudou 11 | Anhui | II | purple | yellow |
| MC274 | Fudou 13 | Anhui | III | purple | yellow |
| MC275 | Fudou 9765 | Anhui | II | purple | yellow |
| MC276 | Fudou 9 hao | Anhui | IV | purple | yellow |
| MC277 | Fuzajiaodou 1 hao | Anhui | III | purple | yellow |
| MC278 | Kelong 188 | Anhui | III | purple | yellow |
| MC279 | Meng 9801 | Anhui | IV | white | yellow |
| MC280 | Suike 15 | Anhui | II | purple | yellow |
| MC281 | Suike 998 | Anhui | IV | purple | yellow |
| MC282 | Wandou 28 | Anhui | IV | purple | yellow |
| MC283 | Wandou 29 | Anhui | IV | white | yellow |
| MC284 | Wandou 30 | Anhui | III | purple | yellow |
| MC285 | Wandou 31 | Anhui | III | purple | yellow |
| MC286 | Wandou 32 | Anhui | III | white | yellow |
| MC287 | Wandou 33 | Anhui | II | purple | yellow |
| MC288 | Wandou 34 | Anhui | III | purple | yellow |
| MC289 | Wandou 35 | Anhui | III | purple | yellow |
| MC290 | Wansu 01-15 | Anhui | III | white | yellow |
| MC291 | Wansu 2156 | Anhui | III | white | yellow |
| MC292 | Wansu 5717 | Anhui | III | purple | yellow |
| MC293 | Wodou 5 hao | Anhui | III | purple | yellow |
| MC294 | Wodou 6 hao | Anhui | III | purple | yellow |
| MC295 | YuanYu 6 hao | Anhui | III | purple | yellow |
| MC296 | Kedou 1 hao | Beijing | IV | purple | yellow |
| MC297 | Kefeng 29 | Beijing | III | purple | yellow |
| MC298 | Shidou 5 hao | Beijing | III | white | yellow |
| MC299 | Zhonghuang 13 | Beijing | III | purple | yellow |
| MC300 | Zhonghuang 30 | Beijing | II | purple | yellow |
| MC301 | Zhonghuang 39 | Beijing | II | white | yellow |
| MC302 | Zhonghuang 40 | Beijing | III | white | yellow |
| MC303 | Zhonghuang 41 | Beijing | III | white | yellow |
| MC304 | Zhonghuang 42 | Beijing | III | purple | yellow |
| MC305 | Zhonghuang 43 | Beijing | III | purple | yellow |
| MC306 | Zhonghuang 44 | Beijing | III | purple | yellow |
| MC307 | Zhonghuang 48 | Beijing | IV | purple | yellow |
| MC308 | Zhonghuang 49 | Beijing | III | purple | yellow |
| MC309 | Zhonghuang 50 | Beijing | III | purple | yellow |
| MC310 | Zhonghuang 51 | Beijing | III | white | yellow |
| MC311 | Zhonghuang 56 | Beijing | II | purple | yellow |
| MC312 | Zhonghuang 57 | Beijing | III | purple | yellow |
| MC313 | Zhonghuang 59 | Beijing | III | white | yellow |
| MC314 | Zhonghuang 62 | Beijing | III | purple | yellow |
| MC315 | Zhonghuang 66 | Beijing | III | purple | yellow |
| MC316 | Zhonghuang 69 | Beijing | III | white | yellow |
| MC317 | Zhonghuang 71 | Beijing | III | purple | yellow |
| MC318 | Zhonghuang 74 | Beijing | III | white | yellow |
| MC319 | Zhonghuang 75 | Beijing | II | purple | yellow |
| MC320 | Zhonghuang 79 | Beijing | III | purple | yellow |
| MC321 | Zhonghuang 901 | Beijing | III | purple | yellow |
| MC322 | Zhongpin 661 | Beijing | II | white | yellow |
| MC323 | Yindou 1 hao | Gansu | II | white | yellow |
| MC324 | Baodou 3 hao | Hebei | III | white | yellow |
| MC325 | Cangdou 10 hao | Hebei | II | purple | yellow |
| MC326 | Cangdou 6 hao | Hebei | I | purple | yellow |
| MC327 | Cangdou 7 hao | Hebei | IV | purple | yellow |
| MC328 | Handou 10 hao | Hebei | III | purple | yellow |
| MC329 | Handou 5 hao | Hebei | II | purple | yellow |
| MC330 | Handou 6 hao | Hebei | III | white | yellow |
| MC331 | Handou 7 hao | Hebei | III | purple | yellow |
| MC332 | Handou 8 hao | Hebei | III | purple | yellow |
| MC333 | Handou 9 hao | Hebei | III | purple | yellow |
| MC334 | Jidou 12 | Hebei | II | purple | yellow |
| MC335 | Jidou 17 | Hebei | III | white | yellow |
| MC336 | Jidou 18 | Hebei | Ⅴ | white | yellow |
| MC337 | Jidou 19 | Hebei | III | white | yellow |
| MC338 | Jidou 20 | Hebei | IV | purple | yellow |
| MC339 | Jidou 21 | Hebei | III | purple | yellow |
| MC340 | Jidou 22 | Hebei | III | white | yellow |
| MC341 | Langdou 6 hao | Hebei | II | purple | yellow |
| MC342 | Nongdadou 2 hao | Hebei | III | white | yellow |
| MC343 | Shidou 1 hao | Hebei | III | purple | yellow |
| MC344 | Shidou 3 hao | Hebei | IV | purple | yellow |
| MC345 | Shidou 4 hao | Hebei | III | purple | yellow |
| MC346 | Shidou 6 hao | Hebei | III | purple | yellow |
| MC347 | Shidou 7 hao | Hebei | III | purple | yellow |
| MC348 | Shidou 8 hao | Hebei | III | white | yellow |
| MC349 | Wuxing 4 hao | Hebei | III | purple | yellow |
| MC350 | Andou 1 hao | Henan | III | purple | yellow |
| MC351 | Andou 4 hao | Henan | IV | purple | yellow |
| MC352 | Dingcun 931 yaoheidou | Henan | IV | white | black |
| MC353 | Fandou 11 | Henan | III | purple | yellow |
| MC354 | Fandou 4 hao | Henan | III | purple | yellow |
| MC355 | Kaidou 41 | Henan | III | purple | yellow |
| MC356 | Pingdou 2 hao | Henan | III | white | yellow |
| MC357 | Pudou 129 | Henan | III | purple | yellow |
| MC358 | Pudou 1802 | Henan | III | purple | yellow |
| MC359 | Pudou 206 | Henan | IV | purple | yellow |
| MC360 | Pudou 857 | Henan | IV | white | yellow |
| MC361 | Pudou 955 | Henan | IV | purple | yellow |
| MC362 | Shangdou 14 | Henan | Ⅴ | purple | yellow |
| MC363 | Shangdou 6 hao | Henan | IV | purple | yellow |
| MC364 | Yudou 22 | Henan | IV | purple | yellow |
| MC365 | Zheng 03-4 | Henan | Ⅴ | purple | yellow |
| MC366 | Zheng 120 | Henan | III | purple | yellow |
| MC367 | Zheng 4066 | Henan | Ⅴ | purple | yellow |
| MC368 | Zheng 7051 | Henan | IV | purple | yellow |
| MC369 | Zheng 9525 | Henan | IV | white | yellow |
| MC370 | Zheng 9805 | Henan | IV | purple | yellow |
| MC371 | Zhengdou 30 | Henan | III | purple | yellow |
| MC372 | Zhoudou 16 | Henan | III | purple | yellow |
| MC373 | Zhoudou 17 | Henan | III | purple | yellow |
| MC374 | Zhoudou 18 | Henan | IV | purple | yellow |
| MC375 | Zhoudou 19 | Henan | IV | purple | yellow |
| MC376 | Zhoudou 20 | Henan | IV | purple | yellow |
| MC377 | Zhoudou 21 | Henan | IV | white | yellow |
| MC378 | Zhoudou 23 | Henan | IV | white | yellow |
| MC379 | Huaidou 11 | Jiangsu | Ⅴ | purple | yellow |
| MC380 | Huaidou 12 | Jiangsu | IV | purple | yellow |
| MC381 | Huaidou 7 hao | Jiangsu | IV | purple | yellow |
| MC382 | Huaidou 9 hao | Jiangsu | III | white | yellow |
| MC383 | Huaixiandou 5 hao | Jiangsu | III | white | green |
| MC384 | Nannong 26 | Jiangsu | Ⅶ | white | black |
| MC385 | Nannong 28 | Jiangsu | Ⅵ | purple | yellow |
| MC386 | Nannong 30 | Jiangsu | Ⅵ | purple | black |
| MC387 | Nannong 32 | Jiangsu | Ⅴ | purple | yellow |
| MC388 | Nannong 33 | Jiangsu | Ⅴ | white | black |
| MC389 | Nannong 34 | Jiangsu | IV | white | yellow |
| MC390 | Nannong 39 | Jiangsu | IV | white | yellow |
| MC391 | Nannong 41 | Jiangsu | Ⅴ | white | yellow |
| MC392 | Nannongcaidou 6 hao | Jiangsu | Ⅵ | white | yellow |
| MC393 | Sidou 520 | Jiangsu | III | purple | yellow |
| MC394 | Sudou 5 hao | Jiangsu | III | purple | yellow |
| MC395 | Sudou 6 hao | Jiangsu | III | purple | yellow |
| MC396 | Sudou 7 hao | Jiangsu | III | purple | yellow |
| MC397 | Sudou 8 hao | Jiangsu | I | white | yellow |
| MC398 | Suxiandou 19 | Jiangsu | Ⅵ | purple | yellow |
| MC399 | Suxiandou 21 | Jiangsu | Ⅶ | purple | yellow |
| MC400 | Suxiandou 22 | Jiangsu | III | purple | yellow |
| MC401 | Tongdou 10 hao | Jiangsu | IV | white | yellow |
| MC402 | Tongdou 2006 | Jiangsu | III | white | yellow |
| MC403 | Tongdou 6 hao | Jiangsu | Ⅵ | purple | green |
| MC404 | Tongdou 7 hao | Jiangsu | Ⅵ | purple | yellow |
| MC405 | Tongdou 8 hao | Jiangsu | Ⅵ | purple | yellow |
| MC406 | Tongdou 9 hao | Jiangsu | Ⅵ | purple | yellow |
| MC407 | Xudou 13 | Jiangsu | III | white | yellow |
| MC408 | Xudou 14 | Jiangsu | III | purple | yellow |
| MC409 | Xudou 16 | Jiangsu | III | purple | yellow |
| MC410 | Xudou 17 | Jiangsu | IV | white | yellow |
| MC411 | Xudou 18 | Jiangsu | III | white | yellow |
| MC412 | Xudou 20 | Jiangsu | IV | white | yellow |
| MC413 | Xudou 21 | Jiangsu | IV | white | yellow |
| MC414 | Canghei 1 hao | Shandong | III | white | black |
| MC415 | Dedou 99-16 | Shandong | III | white | black |
| MC416 | Hedou 12 | Shandong | III | purple | yellow |
| MC417 | Hedou 14 | Shandong | III | white | yellow |
| MC418 | Hedou 15 | Shandong | III | purple | yellow |
| MC419 | Hedou 16 | Shandong | III | white | yellow |
| MC420 | Hedou 18 | Shandong | III | purple | yellow |
| MC421 | Hedou 19 | Shandong | III | purple | yellow |
| MC422 | Hedou 20 | Shandong | III | purple | yellow |
| MC423 | Hedou 21 | Shandong | III | white | yellow |
| MC424 | Hedou 22 | Shandong | III | white | yellow |
| MC425 | Hedou 23 | Shandong | III | purple | yellow |
| MC426 | Lindou 10 hao | Shandong | III | purple | yellow |
| MC427 | Lindou 9 hao | Shandong | IV | white | yellow |
| MC428 | Luhuang 1 hao | Shandong | IV | white | yellow |
| MC429 | Qihuang 33 | Shandong | III | purple | yellow |
| MC430 | Qihuang 34 | Shandong | III | white | yellow |
| MC431 | Qihuang 35 | Shandong | III | white | yellow |
| MC432 | Qihuang 36 | Shandong | III | white | yellow |
| MC433 | Shanning 11 | Shandong | III | white | yellow |
| MC434 | Shanning 14 | Shandong | III | white | yellow |
| MC435 | Shanning 15 | Shandong | II | white | yellow |
| MC436 | Shanning 16 | Shandong | III | white | yellow |
| MC437 | Shanning 17 | Shandong | III | white | yellow |
| MC438 | Shengdou 10 hao | Shandong | IV | purple | yellow |
| MC439 | Shengdou 14 | Shandong | III | white | yellow |
| MC440 | Shengdou 5 hao | Shandong | III | white | yellow |
| MC441 | Shengdou 9 hao | Shandong | III | white | yellow |
| MC442 | Suike 12 | Shandong | IV | white | yellow |
| MC443 | Suike 8 hao | Shandong | III | white | yellow |
| MC444 | Suike 9 hao | Shandong | IV | purple | yellow |
| MC445 | Weidou 10 hao | Shandong | III | white | yellow |
| MC446 | Weidou 7 hao | Shandong | III | purple | yellow |
| MC447 | Weidou 8 hao | Shandong | III | purple | yellow |
| MC448 | Weidou 9 hao | Shandong | III | white | yellow |
| MC449 | Fendou 56 | Shānxi | II | purple | yellow |
| MC450 | Fendou 78 | Shānxi | III | white | yellow |
| MC451 | Fendou 79 | Shānxi | IV | purple | yellow |
| MC452 | Jinda78 | Shānxi | IV | white | yellow |
| MC453 | Jindazaohuang 2 hao | Shānxi | II | white | yellow |
| MC454 | Jindou 25 | Shānxi | I | purple | yellow |
| MC455 | Jindou 37 | Shānxi | IV | white | yellow |
| MC456 | Jindou 39 | Shānxi | IV | white | yellow |
| MC457 | Jinyi 19 | Shānxi | II | purple | yellow |
| MC458 | Yundou 101 | Shānxi | IV | white | yellow |
| MC459 | Baodou 6 hao | Shǎnxi | IV | purple | yellow |
| MC460 | Qindou 11 | Shǎnxi | Ⅴ | purple | yellow |
| MC461 | Qindou 12 | Shǎnxi | IV | white | yellow |
| MC462 | Qindou 13 | Shǎnxi | IV | purple | yellow |
| MC463 | Fudou 71 | Fujian | III | purple | yellow |
| MC464 | Mindou 5 hao | Fujian | III | white | yellow |
| MC465 | Quandou 12 | Fujian | IV | purple | yellow |
| MC466 | Quandou 13 | Fujian | III | white | yellow |
| MC467 | Huachun 1 hao | Guangdong | Ⅵ | purple | yellow |
| MC468 | Huachun 3 hao | Guangdong | III | white | yellow |
| MC469 | Huachun 5 hao | Guangdong | IV | white | yellow |
| MC470 | Huachun 6 hao | Guangdong | III | purple | yellow |
| MC471 | Huachun 8 hao | Guangdong | III | purple | yellow |
| MC472 | Huaxia 1 hao | Guangdong | Ⅵ | white | yellow |
| MC473 | Huaxia 2 hao | Guangdong | Ⅵ | white | yellow |
| MC474 | Huaxia 3 hao | Guangdong | Ⅸ | white | yellow |
| MC475 | Huaxia 5 hao | Guangdong | Ⅴ | purple | yellow |
| MC476 | Huaxia 6 hao | Guangdong | Ⅷ | white | yellow |
| MC477 | Huaxia 9 hao | Guangdong | Ⅷ | white | yellow |
| MC478 | Guichun 10 hao | Guangxi | IV | purple | yellow |
| MC479 | Guichun 11 | Guangxi | IV | purple | yellow |
| MC480 | Guichun 12 | Guangxi | IV | purple | yellow |
| MC481 | Guichun 13 | Guangxi | IV | purple | yellow |
| MC482 | Guichun 15 | Guangxi | IV | white | yellow |
| MC483 | Guichun 8 hao | Guangxi | IV | white | yellow |
| MC484 | Guichundou 103 | Guangxi | Ⅴ | white | yellow |
| MC485 | Guichundou 104 | Guangxi | Ⅴ | white | yellow |
| MC486 | Guichundou 106 | Guangxi | Ⅴ | purple | yellow |
| MC487 | Guixia 5 hao | Guangxi | Ⅷ | purple | yellow |
| MC488 | Guixiadou 105 | Guangxi | Ⅷ | purple | yellow |
| MC489 | Guixiadou 2 hao | Guangxi | Ⅷ | purple | yellow |
| MC490 | Andou 5 hao | Guizhou | IV | purple | yellow |
| MC491 | Andou 7 hao | Guizhou | IV | purple | yellow |
| MC492 | Qiandou 7 hao | Guizhou | IV | purple | yellow |
| MC493 | Qiandou 8 hao | Guizhou | IV | purple | yellow |
| MC494 | Edou 10 hao | Hubei | IV | white | yellow |
| MC495 | Endou 31 | Hubei | III | white | yellow |
| MC496 | Jingdou 2 hao | Hubei | IV | purple | yellow |
| MC497 | Jingdou 3 hao | Hubei | IV | purple | yellow |
| MC498 | Jingdou 4 hao | Hubei | IV | white | yellow |
| MC499 | Tianlong 1 hao | Hubei | III | white | yellow |
| MC500 | Tianlong 2 hao | Hubei | III | white | yellow |
| MC501 | Zhongdou 39 | Hubei | III | white | yellow |
| MC502 | Zhongdou 40 | Hubei | II | white | yellow |
| MC503 | Zhongdou 41 | Hubei | Ⅴ | white | yellow |
| MC504 | Zhongdou 43 | Hubei | Ⅴ | white | yellow |
| MC505 | Xiangchundou 24 | Hunan | II | white | yellow |
| MC506 | Xiangchundou 26 | Hunan | II | white | yellow |
| MC507 | Xiangchundou V7 | Hunan | Ⅵ | white | yellow |
| MC508 | Xiangchundou V8 | Hunan | Ⅵ | white | yellow |
| MC509 | Gandou 6 hao | Jiangxi | IV | purple | yellow |
| MC510 | Jiaoda 02-89 | Shanghai | III | purple | yellow |
| MC511 | Jiaoda 09-5 | Shanghai | III | white | green |
| MC512 | Chengdou 14 | Sichuan | III | white | yellow |
| MC513 | Chengdou 16 | Sichuan | III | white | yellow |
| MC514 | Chengdou 17 | Sichuan | III | white | yellow |
| MC515 | Chuandou 12 | Sichuan | III | purple | yellow |
| MC516 | Chuandou 13 | Sichuan | II | purple | yellow |
| MC517 | Chuandou 14 | Sichuan | II | white | yellow |
| MC518 | Chuandou 15 | Sichuan | III | white | yellow |
| MC519 | Chuandou 16 | Sichuan | III | white | yellow |
| MC520 | Gongdou 18 | Sichuan | IV | white | yellow |
| MC521 | Gongdou 20 | Sichuan | III | white | yellow |
| MC522 | Gongdou 22 | Sichuan | III | white | yellow |
| MC523 | Gongdou 23 | Sichuan | III | white | yellow |
| MC524 | Gongqiudou 4 hao | Sichuan | Ⅷ | purple | yellow |
| MC525 | Gongqiudou 5 hao | Sichuan | Ⅷ | purple | yellow |
| MC526 | Gongqiudou 7 hao | Sichuan | Ⅷ | purple | yellow |
| MC527 | Gongqiudou 8 hao | Sichuan | Ⅸ | white | yellow |
| MC528 | Gongxiadou 10 hao | Sichuan | Ⅵ | white | green |
| MC529 | Gongxiadou 9 hao | Sichuan | Ⅵ | white | yellow |
| MC530 | Nanchundou 28 | Sichuan | III | purple | yellow |
| MC531 | Nanchundou 29 | Sichuan | III | purple | yellow |
| MC532 | Nandou 11 | Sichuan | Ⅵ | white | yellow |
| MC533 | Nandou 12 | Sichuan | Ⅷ | white | yellow |
| MC534 | Nandou 14 | Sichuan | Ⅸ | white | yellow |
| MC535 | Nandou 15 | Sichuan | Ⅸ | white | yellow |
| MC536 | Nandou 18 | Sichuan | Ⅷ | purple | yellow |
| MC537 | Nandou 19 | Sichuan | Ⅷ | white | yellow |
| MC538 | Nandou 22 | Sichuan | Ⅸ | purple | yellow |
| MC539 | Nandou 23 | Sichuan | III | white | yellow |
| MC540 | Nandou 24 | Sichuan | III | purple | yellow |
| MC541 | Nanheidou 20 | Sichuan | Ⅷ | purple | black |
| MC542 | Nanxiadou 25 | Sichuan | Ⅶ | white | yellow |
| MC543 | Nanxiadou 27 | Sichuan | Ⅷ | white | green |
| MC544 | Diandou 4 hao | Yunnan | III | white | yellow |
| MC545 | Diandou 6 hao | Yunnan | IV | white | yellow |
| MC546 | Diandou 7 hao | Yunnan | Ⅴ | white | yellow |
| MC547 | Liqiu 3 hao | Zhejiang | Ⅷ | white | yellow |
| MC548 | Quqiu 3 hao | Zhejiang | Ⅷ | white | yellow |
| MC549 | Quqiu 5 hao | Zhejiang | Ⅷ | purple | green |
| MC550 | Quxian 3 hao | Zhejiang | Ⅵ | white | yellow |
| MC551 | Quxian 5 hao | Zhejiang | Ⅷ | purple | green |
| MC552 | Quxian 6 hao | Zhejiang | Ⅷ | purple | yellow |
| MC553 | Xiaonongqiuyan | Zhejiang | Ⅷ | purple | green |
| MC554 | Zhenong 3 hao | Zhejiang | Ⅵ | white | yellow |
| MC555 | Zhenong 6 hao | Zhejiang | Ⅵ | white | yellow |
| MC556 | Zhenong 8 hao | Zhejiang | Ⅵ | white | yellow |
| MC557 | Yudou 2 hao | Chongqing | IV | white | yellow |
| MC558 | Yudou 3 hao | Chongqing | IV | white | yellow |
| MC559 | Changjiangchun 1 hao | Chongqing | IV | white | yellow |
| MC560 | Changjiangchun 2 hao | Chongqing | IV | white | yellow |

## Supplementary Table 3 Analysis of variance of six agronomic traits in the MCSCP.

| **Source of variation** | **Days to flowering** | | | **Days to maturity** | | | **Main stem node number** | | |
| --- | --- | --- | --- | --- | --- | --- | --- | --- | --- |
|  | **DF** | **MS** | ***F*-value** | **DF** | **MS** | ***F*-value** | **DF** | **MS** | ***F*-value** |
| Cultivar | 559 | 450.09 | 11.04** | 559 | 797.60 | 8.25** | 559 | 39.89 | 10.12** |
| Env | 1 | 5765.16 | 136.59** | 1 | 46616 | 316.36** | 1 | 105.36 | 29.36** |
| Rep (env) | 3 | 3.64 | 1.93 | 3 | 71.17 | 6.44 | 3 | 0.78 | 0.74 |
| Cultivar×Env | 557 | 40.74 | 21.59** | 534 | 95.38 | 8.64** | 549 | 3.93 | 3.72** |
| Error | 1657 | 1.88 |  | 1532 | 11.04 |  | 1596 | 1.06 |  |
| Total | 2777 |  |  | 2629 |  |  | 2708 |  |  |
| **Source of variation** | **100-seed weight** | | | **Seed protein content** | | | **Seed oil content** | | |
|  | **DF** | **MS** | ***F*-value** | **DF** | **MS** | ***F*-value** | **DF** | **MS** | ***F*-value** |
| Cultivar | 557 | 43.71 | 2.72** | 548 | 21.29 | 2.22** | 548 | 21.46 | 2.53** |
| Env | 1 | 4341.49 | 136.85** | 1 | 564.01 | 24.83** | 1 | 556.79 | 32.90** |
| Rep (Env) | 3 | 18.05 | 10.17** | 3 | 15.33 | 17.17** | 3 | 12.82 | 15.47** |
| Geno×Env | 531 | 16.16 | 9.11** | 492 | 9.36 | 10.49** | 492 | 8.27 | 9.98** |
| Error | 1588 | 1.78 |  | 1314 | 8.47 |  | 1314 | 0.22 |  |
| Total | 2680 |  |  | 2358 |  |  | 2358 |  |  |

MCSCP represents the modern Chinese soybean cultivar population.

Env.: environment; Rep: replication.

DF: degree freedom; MS: Mean square; ** indicate significance level at α=0.01.

## Supplementary Table 4. The breeding approaches of the released cultivars in NNC, HHH and SC of MCSCP.

| **Population** | **No. cultivar** | **Breeding approach** | | | | | |
| --- | --- | --- | --- | --- | --- | --- | --- |
|  |  | **H** | **S** | **MH** | **M** | **DNA** | **RS** |
| NNC | 279 | 266 | 4 | 3 | 3 | 1 | 2 |
| HHH | 155 | 144 | 5 |  | 3 |  | 3 |
| SC | 126 | 113 | 6 |  | 7 |  |  |
| MCSCP | 560 | 523 | 15 | 3 | 13 | 1 | 5 |

NNC, HHH and SC represent Northeast and Northwest China, Huang-Huai-Hai Valleys and Southern China, respectively. MCSCP represents the modern Chinese soybean cultivar population.

H: hybridization breeding; S: natural variant selection; MH: hybridization combined with mutation; M: mutation breeding; DNA: DNA-transferring; RS: recurrent selection.

## Supplementary Table 5. The used times and types of the immediate parents in the development of MCSCP.

| **Population** | **Frequency** | | | | |
| --- | --- | --- | --- | --- | --- |
|  | **1** | **2-5** | **6-10** | **11** | **13** |
| NNC | 257 (257) | 94 (247) | 5 (39) | 1 (11) |  |
| HHH | 154 (154) | 44 (119) | 5 (33) |  |  |
| SC | 141 (141) | 33 (78) | 2 (14) |  | 1 (13) |
| MCSCP | 532 (532) | 169 (444) | 15 (106) | 1 (11) | 1 (13) |
| **Population** | **The type of immediate parents** | | | | |
|  | **Landrace** | **Foreign variety** | **Cultivar** | **Breeding line** | **Total** |
| NNC | 6 (6) | 23 (29) | 141 (291) | 187 (228) | 357 (554) |
| HHH | 7 (9) | 16 (17) | 86 (168) | 94 (112) | 203 (306) |
| SC | 28 (39) | 14 (24) | 59 (96) | 76 (87) | 177 (246) |
| MCSCP | 41 (54) | 50 (70) | 273 (555) | 354 (427) | 718 (1106) |

NNC, HHH and SC represent Northeast and Northwest China, Huang-Huai-Hai Valleys and Southern China, respectively. MCSCP represents the modern Chinese soybean cultivar population.

Outside of the parentheses is the number of immediate parents, and in parentheses is the sum of used times of the immediate parents.

## Supplementary Table 6. Name list of the 41 core-terminal ancestors in MCSCP.

| **Code** | **Region** | **Name of core-terminal ancestor** | **Code** | **Region** | **Name of core-terminal ancestor** |
| --- | --- | --- | --- | --- | --- |
| CA-01 | NNC | Baimei | CA-22 | HHH | Pixianluantiaozhi |
| CA-02 | NNC | Jinyuan | CA-23 | HHH | Qinyangshuibaidou |
| CA-03 | NNC | Silihuang | CA-24 | HHH | Tiejiaohuang |
| CA-04 | NNC | Keshansilijia | CA-25 | HHH | Yidupingdinghuang |
| CA-05 | NNC | Duludou | CA-26 | SC | Vegetable soybean population in Wuhan |
| CA-06 | NNC | Tiejiazi | CA-27 | SC | Fengxiansuidaohuang |
| CA-07 | NNC | Xiongyuexiaohuangdou | CA-28 | SC | 51-83 |
| CA-08 | NNC | Tiejiasilihuang | CA-29 | SC | Huliuyuebai |
| CA-09 | NNC | Xiaolidou 9 hao | CA-30 | SC | Pudongdahuangdou |
| CA-10 | NNC | Yongfengdou | CA-31 | SC | Zanbian 20 |
| CA-11 | NNC | Suoyiling | CA-32 | SC | Zigongqingpidou |
| CA-12 | NNC | Xiaolihuang | CA-33 | SC | Putiandahuangdou |
| CA-13 | NNC | Xunkedangdizhong | CA-34 | F | Shishengchangye |
| CA-14 | NNC | Xiaojinhuang | CA-35 | F | A.K. |
| CA-15 | HHH | Binhaidabaihua | CA-36 | F | Lincoln |
| CA-16 | HHH | Tongshantianedan | CA-37 | F | Richland |
| CA-17 | HHH | Dingtaopingdingdahuangdou | CA-38 | F | Dunfield |
| CA-18 | HHH | Huaxiandalvdou | CA-39 | F | Mandarin |
| CA-19 | HHH | Shouzhangdifangzhong | CA-40 | F | Mammoth Yellow |
| CA-20 | HHH | Jimoyoudou | CA-41 | F | Otootan |
| CA-21 | HHH | Lusijiaoqi |  |  |  |

NNC, HHH and SC represent Northeast and Northwest China, Huang-Huai-Hai Valleys and Southern China, respectively. F represents terminal ancestors from abroad. MCSCP represents the modern Chinese soybean cultivar population.

## Supplementary Table 7. The genetic contribution of terminal ancestor types used in NNC, HHH and SC of the MCSCP.

| **Population** | **No. of cultivars** | **No. of terminal ancestors** | **Core-terminal ancestors** | | **NGC** | | **CGC** | |
| --- | --- | --- | --- | --- | --- | --- | --- | --- |
|  |  |  | **Number** | **%** | **Value** | **%** | **Value** | **%** |
| NNC | 279 | 321 | 33 | 10.28 | 140.42 | 50.33 | 165 | 59.16 |
| HHH | 155 | 231 | 33 | 14.29 | 78.26 | 50.49 | 94 | 60.68 |
| SC | 126 | 222 | 39 | 17.57 | 30.68 | 24.35 | 46 | 36.50 |
| MCSC | 560 | 604 | 41 | 6.79 | 249.03 | 44.47 | 305 | 54.47 |

NNC, HHH and SC represent Northeast and Northwest China, Huang-Huai-Hai Valleys and Southern China, respectively. MCSCP represents the modern Chinese soybean cultivar population.

NGC: nuclear genetic contribution; CGC: cytoplasmic genetic contribution.

## Supplementary Table 8. COP and CGS cross-distribution in the MCSCP.

|  | **COP (×100)** | | | | | | | | | | **Total** |
| --- | --- | --- | --- | --- | --- | --- | --- | --- | --- | --- | --- |
|  | **Range** | **0-0.0** | **0.0-2.5** | **2.5-5.0** | **5.0-7.5** | **7.5-10.0** | **10.0-25.0** | **25.0-50.0** | **50.0-75.0** | **75.0-100** |  |
| CGS  **(×100)** | 74.0-79.0 | 1174 | 367 | 3 |  |  |  |  |  |  | 1544 |
|  | 79.0-84.0 | 8773 | 3823 | 758 | 287 | 97 | 72 | 18 | 1 | 1 | 13830 |
|  | 84.0-89.0 | 65016 | 30371 | 3573 | 1289 | 443 | 556 | 193 | 6 | 5 | 101452 |
|  | 89.0-94.0 | 12820 | 16261 | 5209 | 2158 | 1012 | 1349 | 373 | 21 | 2 | 39205 |
|  | 94.0-99.0 | 90 | 116 | 65 | 34 | 37 | 86 | 56 | 4 | 1 | 489 |
|  | Total | 87873 | 50938 | 9608 | 3768 | 1589 | 2063 | 640 | 32 | 9 | 156520 |

MCSCP represents the modern Chinese soybean cultivar population released in 2006-2015.

COP is the coefficient of parentage in the MCSCP, the actual values were multiplied by 100 in the table.

CGS is the coefficient of genetic similarity in the MCSCP, the actual values were multiplied by 100 in the table.
